# Uncovering hidden disease patterns by simulating clinical diagnostic processes

**DOI:** 10.1038/s41598-018-20826-y

**Published:** 2018-02-05

**Authors:** Abolfazl Ramezanpour, Alireza Mashaghi

**Affiliations:** 1 Leiden Academic Centre for Drug Research, Faculty of Science, Leiden University, Leiden, The Netherlands; 2 Department of Physics, University of Neyshabur, Neyshabur, Iran

## Abstract

Choosing a sequence of observations (often with stochastic outcomes) which maximizes the information gain from a system of interacting variables is essential for a wide range of problems in science and technology, such as clinical diagnostic problems. Here, we use a probabilistic model of diseases and signs/symptoms to simulate the effects of medical decisions on the quality of diagnosis by maximizing an appropriate objective function of the medical observations. The study provides a systematic way of proposing new medical tests, considering the significance of diseases and cost of the suggested observations. The efficacy of methods and role of the objective functions as well as initial signs/symptoms are examined by numerical simulations of the diagnostic process by exhaustive or Monte Carlo sampling algorithms.

## Introduction

Clinical diagnosis is typically made through a process that starts with identifying initial findings and noting the past medical history of the patient and ends with a diagnosis or unresolved differential diagnoses^1,2^. In practice, the sequence of steps one clinician follows may be very different from those taken by another clinician, and the same clinician may approach the problem differently in two nearly identical cases^3^. This variability in diagnostic approach has a complex source and is rooted in the limited and varied extent of knowledge of the clinicians, stochasticity of the decision-making process, and lack of solid risk and cost assessment strategies among others.

Since the classic paper by Ledley and Lusted^4^ where they first detailed on how logic and probabilistic reasoning form the backbone of medical reasoning, there has been much progress in the development of diagnostic decision support systems (DDSS)^5–11^. In recent decades, due to limited availability of appropriate clinical data, there has been growing interest in developing heuristic formal and rigorous mathematical models. These studies covered a wide range of approaches from simple Bayesian models to Bayesian belief networks and neural networks^12–18^.

Here, using simple and rigorous models, we look for determinants of the efficiency of a diagnostic approach, i.e. choice of a sequence of events that leads to a diagnosis. The study involves concepts and tools of machine learning and inference, as well as stochastic optimization, to deal with the model construction and the stochastic nature of the problem^19–22^. In ref.^23^ we used techniques from statistical physics of disordered systems to study this problem with more emphasis on the role of the interaction graph of signs (hereafter, we refer to symptoms or signs as “signs” for simplicity) and diseases in the quality of diagnosis^24–28^. Our models are indeed natural generalizations of the simpler probabilistic models studied in previous works^13–15^, which usually assume that only one disease is behind the findings (exclusive diseases assumption) or the diseases act independently on the signs (causal independence assumption). Moreover, for computational simplicity, it is usually assumed that there is no disease-disease and sign-sign interactions. We showed that such interactions can significantly improve the accuracy of diagnosis without resorting to the exclusive diseases or the causal independence assumption. In this paper, we extend our previous study by introducing new performance measures and optimization algorithms with more focus on the role of the objective function and initial number of observations in the performance of the diagnostics algorithms.

**Figure 1:**
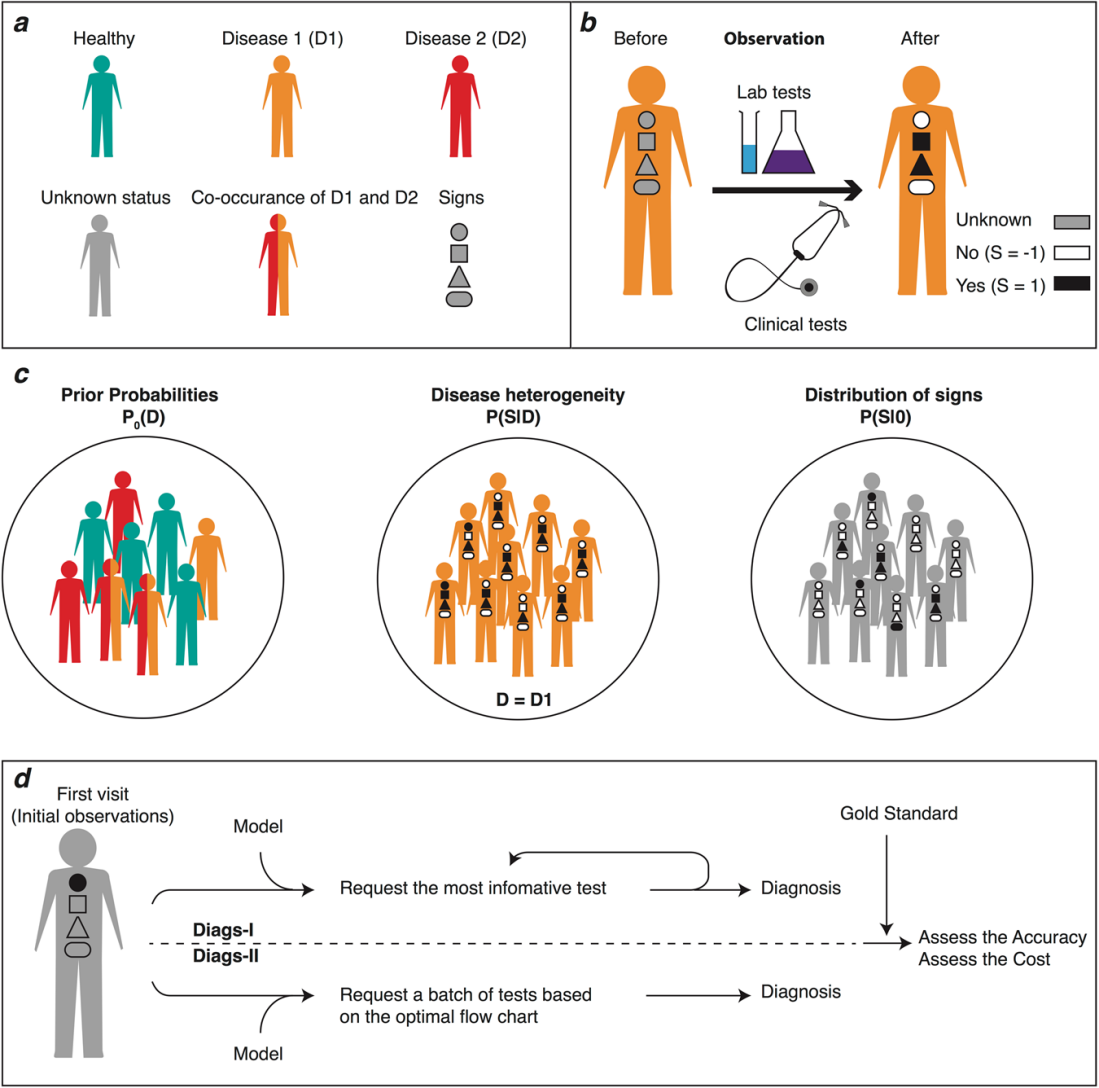
An illustration of the model definitions and the diagnostic processes. (**a**) A patient is represented with a disease pattern **D** (with **0** for the healthy state) and signs **S**. (**b**) A medical test changes an unobserved sign to an observed one with values ±1. (**c**) The probabilistic model is defined with the prior disease probabilities *P*_0_(**D**) and the conditional sign probabilities *P*(**S**|**D**). The leak probability *P*(***S***|**0**) takes into account the effects of unknown or ignored diseases. (**d**) The two diagnostic procedures (Diags-I and Diags-II) start from the same initial findings, but differ in the way the new observations are decided. In Diags-I, the true value of an observed sign is revealed by a medical test before going to the next observation. In Diags-II, the whole process is simulated with the sign values that are inferred from the probabilistic model.

Given a model of disease and sign variables, we aim to propose an optimal sequence of medical tests maximizing an appropriate objective function of the observations (Fig. 1). Here, besides the nature of the model, the structure of the objective function and the initial number and quality of medical tests play a significant role. A reasonable objective function for these kind of problems is provided by the maximum value of the disease likelihood^29^. To reduce the diagnosis time and the mortality and morbidity of diseases, we propose an objective function which gives more weight to the more polarizing observations and dangerous diseases. We see how the initial number of observations and the cost of medical tests in the objective function affect the diagnosis performances in numerical simulations of the models. We also devise an approximate optimization algorithm based on the Monte Carlo sampling to construct an optimal sequence of medical tests for observation.

## Main definitions and problem statement

### Models

Consider a set of *N*_*D*_ binary variables **D** = {*D*_*a*_ = 0, 1: *a* = 1, …, *N*_*D*_}, where *D*_*a*_ = 0,1 shows the absence or presence of disease *a*. We have another set of *N*_*S*_ binary variables **S** = {*S*_*i*_ =± 1: *i* = 1, …, *N*_*S*_} to show the values of sign variables (clinical and laboratory findings). We take *W*_*a*_ for the weight or importance of disease *a*, and *C*_*i*_ for the cost of observing sign *i*. In the following, the weights *W*_*a*_ ∈ (0, 1) and costs *C*_*i*_ ∈ (0, 1) are independent and identically distributed random variables with a uniform probability distribution. The joint probability distribution of the sign and disease variables (i.e., the model) is identified by *P*(**S**; **D**) = *P*(**S**|**D**)*P*_0_(**D**). Here *P*_0_(**D**) is the prior probability distribution of diseases, which could depend on the patient’s characteristics such as gender and age and disease properties such as duration of a disease, mortality rate and transmission rate among others.

Let *P*_*true*_(**S**|**D**) be the true probability distribution of sign variables given disease hypothesis **D**. In practice, we may have access only to a small subset of marginal probabilities of this true distribution. For instance, suppose we are given sign probabilities *P*_*true*_(*S*_*i*_|nodisease), *P*_*true*_(*S*_*i*_, *S*_*j*_|only*D*_*a*_), and *P*_*true*_(*S*_*i*_, *S*_*j*_|only*D*_*a*_, *D*_*b*_) conditioned on the absence of any of the diseases, and the presence of only one and two diseases, respectively. Using the maximum entropy principle^30^, for the conditional probability distribution of signs we take^23^

1 $$P({\bf{S}}|{\bf{D}})=\frac{1}{Z({\bf{D}})}{\varphi }_{0}({\bf{S}})\times \prod _{a}{\varphi }_{a}({\bf{S}}|{D}_{a})\times \prod _{a < b}{\varphi }_{ab}({\bf{S}}|{D}_{a},{D}_{b}),$$

where the partition function *Z*(**D**) is obtained from normalization ∑_**S**_*P*(**S**|**D**) = 1. More precisely, the disease interaction factors (*ϕ*_0_, *ϕ*_*a*_, *ϕ*_*ab*_), are given by

2 $${\varphi }_{0}({\bf{S}})\equiv {e}^{{\sum }_{i}{K}_{i}^{0}{S}_{i}},$$

3 $${\varphi }_{a}({\bf{S}}|{D}_{a})\equiv {e}^{{D}_{a}[{\sum }_{i}{K}_{i}^{a}{S}_{i}+{\sum }_{i < j}{K}_{ij}^{a}{S}_{i}{S}_{j}]},$$

4 $${\varphi }_{ab}({\bf{S}}|{D}_{a},{D}_{b})\equiv {e}^{{D}_{a}{D}_{b}[{\sum }_{i}{K}_{i}^{ab}{S}_{i}+{\sum }_{i < j}{K}_{ij}^{ab}{S}_{i}{S}_{j}]}.$$

**Figure 2:**
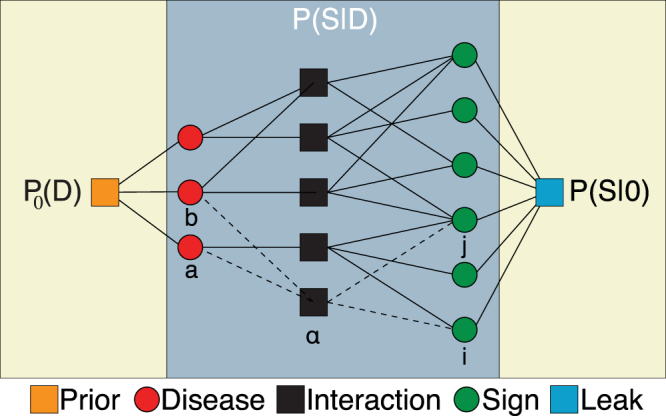
The interaction graph of disease variables (left circles) and sign variables (right circles) related by *M*_*a*_ one-disease and *M*_*ab*_ two-disease interaction factors (middle squares) in addition to interactions induced by the leak probability (right square) and the prior probability of diseases (left square). In general, an interaction factor *α* = *a*, *ab* is connected to *k*_*α*_ signs and *l*_*α*_ diseases^23^.

In principle, the above information from the true probability distribution is sufficient to determine the model parameters ${K}_{i}^{0},{K}_{i}^{a,ab}$, and ${K}_{ij}^{a,ab}$. Figure 2 shows the interaction graph of sign and disease variables related by the above interaction factors^23^. We use *M*_*a*_ and *M*_*ab*_ for the number of one-disease and two-disease interaction factors, respectively. An interaction factor is connected to *k*_*a*_ or *k*_*ab*_ signs depending on the number of involved diseases.

#### Simplifying Assumptions

For simplicity, in the main text, we ignore the sign-sign interactions in the interaction factors (${K}_{ij}^{a}={K}_{ij}^{ab}=0$). That is, we consider the one-disease-one-sign (D1S1) model with parameters ${K}_{i}^{0},{K}_{i}^{a}$, and two-disease-one-sign (D2S1) model with parameters ${K}_{i}^{0},{K}_{i}^{a},{K}_{i}^{ab}$. This allows us to compute exactly the partition function for these models. Moreover, given the true marginals, the parameters ${K}_{i}^{0},{K}_{i}^{a,ab}$ of the D1S1 and D2S1 models can be computed exactly. To be specific, in the main text we focus on the D2S1 model, which works well as long as the number of present diseases in the hypothesis, |**D**|, is less than or equal to two^23^. We shall briefly discuss the results obtained from the simpler D1S1 model and the more difficult D2S2 model (including the two-sign interactions) in Supplementary Information, Appendixes A and B, respectively.

In addition, we assume that the prior disease probability is factorized, ${P}_{0}({\bf{D}})={\prod }_{a=1}^{{N}_{D}}{P}_{0}({D}_{a})$, with ${P}_{0}({D}_{a})={e}^{{K}_{a}^{0}{D}_{a}}/(1+{e}^{{K}_{a}^{0}})$. The parameters ${K}_{a}^{0}$ can be used to control the expected number of present diseases in the hypothesis. For instance, ${K}_{a}^{0}$ can be chosen such that *N*_*D*_*P*_0_(*D*_*a*_ = 1) = |**D**|. Alternatively, we can fix the expected number of disease probabilities which are greater than a threshold value. As long as the number of signs and diseases is small (e.g., *N*_*S*_ = 20, *N*_*D*_ = 5), we work with a fully connected model of the variables, where all the one-disease and two-disease interaction factors (*ϕ*_*a*_, *ϕ*_*ab*_) including the interactions with all the sign variables could be present in the model. The graph parameters defining the structure of a fully connected model are: *M*_*a*_ = *N*_*D*_, *M*_*ab*_ = 0, *k*_*a*_ = *N*_*S*_ in the D1S1 model and *M*_*a*_ = *N*_*D*_, *M*_*ab*_ = *N*_*D*_(*N*_*D*_ − 1)/2, *k*_*a*_ = *k*_*ab*_ = *N*_*S*_ in the D2S1 model. For larger number of variables, we limit ourselves to sparsely connected graphs with smaller number of interaction factors (*M*_*a*_, *M*_*ab*_) and connectivities (*k*_*a*_, *k*_*ab*_).

### Diagnosis

Let us assume that a subset ${{\bf{I}}}_{0}=\{{i}_{1},{i}_{2},\ldots ,{i}_{{N}_{O}}\}$ of the sign variables is observed with values **S**^*o*^. The possible values for the remaining subset of unobserved signs are denoted by **S**^*u*^. At each time step *t* = 1, 2, …, *T* we use a strategy to choose one of the unobserved signs *j*_*t*_ for observation. The sequence of observed signs at time step *t* is represented by **O**(*t*) = **I**_0_∪{*j*_1_, …, *j*_*t*_}. We use **U**(*t*) for the subset of unobserved signs.

At each step we have the disease and sign probabilities,

5 $$P({\bf{D}}|{{\bf{S}}}^{o})\propto \sum _{{{\bf{S}}}^{u}}P({\bf{S}}|{\bf{D}}){P}_{0}({\bf{D}}),$$

6 $$P({{\bf{S}}}^{u}|{{\bf{S}}}^{o})\propto \sum _{{\bf{D}}}P({\bf{S}}|{\bf{D}}){P}_{0}({\bf{D}}),$$

which can be used to compute the disease marginal probabilities *P*(*D*_*a*_|**S**^*o*^) and the sign marginal probabilities *P*(*S*_*i*_|**S**^*o*^). The maximum likelihood (ML) hypothesis **D**^*ML*^ is obtained by maximizing the disease likelihood^29^,

7 $$ {\mathcal L} ({\bf{D}}|{{\bf{S}}}^{o})\equiv \sum _{{{\bf{S}}}^{u}}P({\bf{S}}|{\bf{D}}){P}_{0}({\bf{D}}\mathrm{)}.$$

At each step *t* we choose an unobserved sign for observation which maximizes an appropriate objective function of the chosen sign. A reasonable objective function is the maximum value of the disease likelihood,

8 $${\rm{ML}}(t)\equiv \frac{1}{|{\bf{O}}(t)|}{\langle \mathrm{log} {\mathcal L} ({{\bf{D}}}^{ML}|{{\bf{S}}}^{o}(t))\rangle }_{O}.$$

The average 〈⋅〉_*O*_ in the above equations is taken over the probability distribution of observation outcomes. Note that before the medical observation we only know the marginal probability of the chosen sign *P*(*S*_*j*_|**S**^*o*^(*t* − 1)). And, after each observation (medical test), we obtain the true value of the observed sign.

We assume that the aim of the diagnostic process is to reach the correct diagnosis with the minimum number of medical tests. Obviously, a disease probability that is closer to zero or one could be more helpful to decide if the disease is absent or present. Therefore, we may at each step choose the sign that results to the largest polarization of the disease probabilities:

9 $${\rm{DP}}(t)\equiv \frac{1}{\sum _{a}{W}_{a}}\sum _{a}{W}_{a}{\langle |P({D}_{a}\mathrm{=1}|{{\bf{S}}}^{o}(t))-\frac{1}{2}|\rangle }_{O}.$$

Other measures of polarization, e.g., the root-mean-square of single-disease polarizations, may work as well^23^. Here we are taking into account also the importance or weight of the diseases *W*_*a*_, which could be high for example for life threatening diseases. The *P*(*D*_*a*_ = 1|**S**^*o*^(*t*)) give the disease probabilities after the *t*-th observation. The marginal probabilities are obtained from the reconstructed models of the true probability distribution.

In this paper, however, we are interested in simulation of the above sequential process of decisions and observation for *T* steps, without asking for any real medical test to reveal the true sign values. In other words, we are interested in extrapolation or prediction of the diagnostic process starting from a small subset **I**_0_ of the observed signs and a simple model of the sign and disease variables. Here, an observed sign *j* in the process is treated as a stochastic variable with a value that is sampled from the associated marginal probability *P*(*S*_*j*_|**S**^*o*^(*t* − 1)) at that time step. For brevity, we call this type of diagnosis Diags-II, and Diags-I is used to refer the diagnostic process in which the true sign value is known (by medical test) just after choosing the sign for observation.

More precisely, in the case of Diags-I, at each time step *t* we choose an unobserved sign *j*_*t*_, which maximizes the following objective function

10 $$ {\mathcal E} (t)\equiv {\rm{ML}}(t)+{\lambda }_{P}{\rm{DP}}(t)-{\lambda }_{C}SC(t).$$

Then we do the medical test to find out the true value of the chosen sign, and go to the next step of the diagnostic process. We have included also the sign cost $SC(t)\equiv {C}_{{j}_{t}}$ into the objective function. The *λ*_*P*_ and *λ*_*C*_ are parameters to control the degree of disease polarization and cost of the observations, respectively. In the case of Diags-II, we choose an optimal sequence of decisions **O**(*T*), which maximizes the following objective functional of the candidate observations:

11 $$ {\mathcal E} [{\bf{O}}(T)]\equiv \sum _{t=1}^{T}{\rm{ML}}(t)+{\lambda }_{P}\sum _{t=1}^{T}{\rm{DP}}(t)-{\lambda }_{C}\sum _{t\mathrm{=1}}^{T}SC(t\mathrm{)}.$$

#### Simplifying Assumptions

A greedy approximation of Diags-II is obtained by splitting the whole process into *T* independent steps; this is very similar to Diags-I except the fact that here we do not know the true sign values. But, we have an estimate of the marginal sign probabilities *P*(*S*_*j*_|**S**^*o*^), which can be utilized to assign a good value to the “observed” sign. The time complexity of the optimization algorithm is then of order (*N*_*S*_ − *N*_*O*_)*T* times the complexity of computing the marginal sign/disease probabilities and the maximum likelihood. These computations can be done by approximate inference and optimization algorithms based on the Monte Carlo sampling. For sparse interaction graphs of sign and disease variables, the time complexity of such an algorithm would be proportional to *N*_*S*_.

To simplify the study and reduce the computation time, we shall replace the average over the possible realizations of the observation outcome with the most probable value. Suppose that we are to observe sign *j* at time step *t*. Then, we assume that the outcome of each observation is given by the value which maximizes the corresponding marginal probability at that time step, i.e., *S*_*j*_ = arg max *P*(*S*_*j*_|**S**^*o*^).

### Diagnostic Performance Measures

The main question of this study is: How close are the predictions obtained by Diags-II to the (more expensive) Diags-I? And, when we can trust the outcome of such a diagnostic process? More precisely, given the model of sign and disease variables, we shall see how predictions of the Diags-II improve by increasing the number of initial observations. This of course depends on the quality of the reconstructed models, the structure of the objective function and performance of the optimization algorithm which is used in the study of Diags-II, and the number of initial observed signs.

To check the quality of our extrapolation, we shall take a simple benchmark model for the true probability distribution *P*_*true*_(**S**|**D**). Given, any disease hypothesis **D**^*true*^, the associated signs **S**^*true*^ can then be obtained by taking the most probable signs from the true probability distribution. To be specific, for the true model we take the following exponential distribution:

12 $${P}_{true}({\bf{S}}|{\bf{D}})=\frac{1}{{Z}_{true}({\bf{D}})}{e}^{-H({\bf{S}},{\bf{S}}({\bf{D}}))},$$

where the Hamming distance *H*(**S**, **S**′) = ∑_*i*_(*S*_*i*_ − *S*′_*i*_)^2^/4 gives the number of different signs in the two sign configurations. Here **S**(**D**) defines the signs attributed to **D**. We will choose these signs randomly and uniformly from the configuration space of sign variables.

Consider the diagnostic process for a patient with true disease values **D**^*true*^. At any time step *t*, we compute the overlap of the disease probabilities with the true disease hypothesis,

13 $$DL(t)\equiv \frac{1}{\sum _{a}{W}_{a}}\sum _{a}{W}_{a}\mathrm{(2}{D}_{a}^{true}-\mathrm{1)}(P({D}_{a}=\mathrm{1|}{{\bf{S}}}^{o}(t))-\frac{1}{2}).$$

This shows how well the inferred disease probabilities are close to the true disease values. Obviously, *DL*(*t*) always increases (on average) with the number of observations in the Diags-I. But this quantity can decrease or increase with *t* depending on number of initial observations *N*_*O*_(0).

Another interesting quantity is the first diagnosis time for a specific subset of diseases **A**; we define the first right diagnosis time *T*_*R*_ as the first time at which we find:

*P*(*D*_*a*_ = 1|**S**^*o*^(*t*)) ≥ *P*_*th*_ for at least one of the diseases *a* ∈ **A**.

In the same way we define the first wrong diagnosis time *T*_*W*_ as the first time at which:

*P*(*D*_*a*_ = 1|**S**^*o*^(*t*)) ≥ *P*_*th*_ for at least one of the diseases *a* ∉ **A**.

Then, the probability of having right or wrong diagnosis after *t* observations would critically depend for example on the initial number of observations.

#### Simplifying Assumptions

To obtain an upper bound for the critical number of initial observations, we use a random strategy for suggesting the observations in the diagnostic process. By the random strategy we mean that at each step we choose randomly an unobserved sign for the next observation. Then, in the Diags-I (random), we do a real observation to find out the true value of the chosen sign. Instead, in the Diags-II (random), we assign the most probable value of the sign to the suggested sign for observation and go ahead without doing any real observation.

## Approximation Algorithms

### A (zero-temperature) Monte Carlo algorithm

In the following, we shall work with the sequence configuration **I**_1 → *T*_ ≡ {*j*_1_, …, *j*_*T*_} instead of the whole set of observations **O**(*T*) = **I**_0_∪{*j*_1_, …, *j*_*T*_}. For any such configuration, we can compute the marginal probabilities *P*(*S*_*j*_|**S**^*o*^(*t*)) and *P*(*D*_*a*_|**S**^*o*^(*t*)), and the objective function ${\mathcal E} [{{\bf{I}}}_{1\to T}]$, by an exact algorithm (for small number of variables) or an approximate algorithm (for larger number of variables). In either case, we have to run the algorithm for *T* times to compute the disease probabilities conditioned on the values of the observed signs in the previous steps. Thus, the time complexity of the algorithm is proportional to *T* times the time complexity of computing the objective function^31^. The main steps of the optimization algorithm are:

- Input: the model *P*(**S**; **D**), the weights *W*_*a*_ and costs *C*_*i*_, the parameters *λ*_*P*,*C*_, initial set of observed signs **I**_0_, time steps *T*
- Start from an initial (random) sequence of observations **I**_1→*T*_ = {*j*_1_, …, *j*_*T*_}:
  - compute the objective function ${\mathcal E} [{{\bf{I}}}_{1\to T}]$

- For $$n=\mathrm{1,}\,2,\ldots ,\,{n}_{max}$$:
  - suggest a new configuration **I**′_1→*T*_
  - compute the change ${\rm{\Delta }} {\mathcal E} = {\mathcal E} [{{\bf{I}}{\boldsymbol{^{\prime} }}}_{1\to T}]- {\mathcal E} [{{\bf{I}}}_{1\to T}]$ in the objective function
  - accept the new configuration if ${\rm{\Delta }} {\mathcal E}  > 0$

- Output: the (local) optimal configuration ${{\bf{I}}}_{1\to T}^{opt}$

Computing the objective function and generating a new sequence configuration **I**′_1 →*T*_ from **I**_1→*T*_ are the main parts of the algorithm. In a previous study^23^, we found that a good heuristic strategy is to choose at each step the most positive unobserved sign for the next observation. The most positive sign is the one with the maximum probability of being positive, that is ${i}_{MP}={\rm{\arg }}\,{{\rm{\max }}}_{i\in U}P({S}_{i}=+\mathrm{1|}{{\bf{S}}}^{o})$. It is important that the assigned values are as close as possible to the true values. By choosing the most positive signs we indeed try to reduce the error in prediction of the values of the observed signs in the simulation process. Note that a wrong assignment at the early stages of the process can significantly affect the whole process, consequently affecting the diagnosis.

Here we use this finding to guide the updating step of the optimization algorithm. More specifically, we use the following rules to update a sequence configuration **I**_1→*T*_:

- choose randomly a time step 1 ≤ *τ* ≤ *T*
- for *t* = *τ*,…*T*, suggest an unobserved sign *j*_*t*′_ with a probability proportional to $P({S}_{{j}_{t^{\prime} }}=+\mathrm{1|}{{\bf{S}}}^{o}(t-1))$

The above process suggests the new sequence **I**′_1→*T*_ = {*j*_1_, …, *j*_*τ*−1_, *j*_*τ*_′, …, *j*′_*T*_} which is accepted only if the new sequence increases the objective function. The success probability of a candidate sequence suggested in this way is about 0.57 (in 660 trials) for the Diags-II, with *N*_*S*_ = 500, *N*_*D*_ = 50, *N*_*O*_(0) = 50, and *T* = 50. Details of computing the objective function is given in Methods Section. Very briefly, to compute the objective function we need the sign and disease marginal probabilities (for *DP*(*t*)), which are estimated by a standard Monte Carlo algorithm, and the maximum likelihood value (for *ML*(*t*)), which is estimated by a Simulated Annealing algorithm^25^. The latter computation can again be done by a zero-temperature Monte Carlo algorithm, but since it determines the objective function we prefer to employ a more accurate optimization algorithm. The time complexity of these algorithms in a sparse D2S1 model is proportional to the number of diseases.

## Results

**Figure 3:**
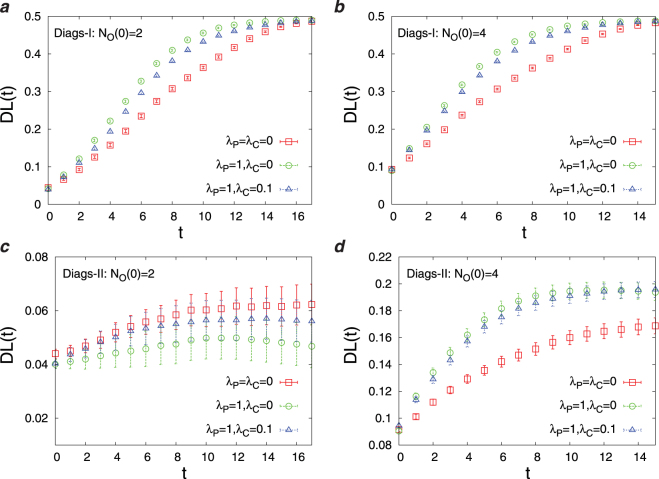
Dependence of *DL*(*t*) on the initial number of observed signs *N*_*O*_(0) and the parameters *λ*_*P*_, *λ*_*C*_. The results have been obtained from the D2S1 model by (**a**)–(**b**) the Diags-I (greedy), and (**c**)–(**d**) the Diags-II (greedy) with the prior probabilities *P*_0_(*D*_*a*_ = 1) = 2/*N*_*D*_. The model parameters of the (fully connected) D2S1 model are obtained exactly from the conditional marginals of the true exponential model. A disease hypothesis is chosen randomly for the simulation with a probability proportional to the weights of the present diseases. All the marginal probabilities have been computed exactly for a small number of sign and disease variables (*N*_*S*_ = 20 and *N*_*D*_ = 5). The data are results of averaging over at least 500 independent realizations of the models and simulation process.

**Figure 4:**
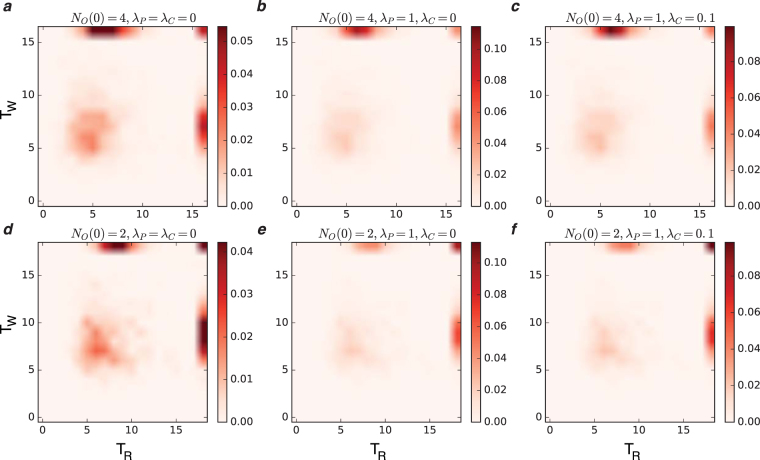
The joint probability of the first diagnosis times, *P*(*T*_*R*_, *T*_*W*_), for different numbers of initial observations *N*_*O*_(0) and the parameters *λ*_*P*_, *λ*_*C*_. The panels display the cases: (**a**)–(**c**) *N*_*O*_(0) = 4 and (**d**–**f**) *N*_*O*_(0) = 2 for (*λ*_*P*_ = *λ*_*C*_ = 0), (*λ*_*P*_ = 1, *λ*_*C*_ = 0), (*λ*_*P*_ = 1, *λ*_*C*_ = 0.1), respectively. The results have been obtained from the D2S1 model by the Diags-II (greedy) with the prior probabilities *P*_0_(*D*_*a*_ = 1) = 2/*N*_*D*_, and the threshold probability *P*_*th*_ = 0.9. The last values of *T*_*R*_ and *T*_*W*_ are reserved for the case in which the corresponding disease probabilities remain less than the threshold value during the whole process. The model parameters of the (fully connected) D2S1 model are obtained exactly from the conditional marginals of the true exponential model. A disease hypothesis is chosen randomly for the simulation with a probability proportional to the weights of the present diseases. The number of present diseases in the hypothesis is |**D**| = 2. All the marginal probabilities have been computed exactly for a small number of sign and disease variables (*N*_*S*_ = 20 and *N*_*D*_ = 5). The data are results of at least 500 independent realizations of the model and simulation process.

**Figure 5:**
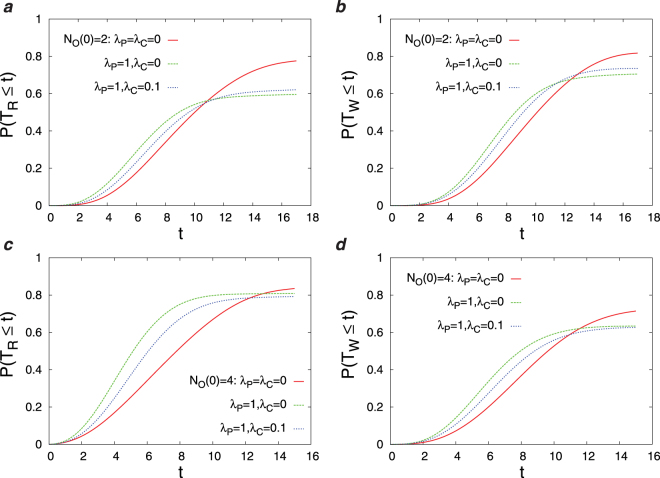
The cumulative probabilities *P*(*T*_*R*_ ≤ *t*) and *P*(*T*_*W*_ ≤ *t*) of the first diagnosis times for different numbers of the initial observations *N*_*O*_(0) and the parameters *λ*_*P*_, *λ*_*C*_. The results have been obtained from the D2S1 model by the Diags-II (greedy) with the prior probabilities *P*_0_(*D*_*a*_ = 1) = 2/*N*_*D*_, and the threshold probability *P*_*th*_ = 0.9. The model parameters of the (fully connected) D2S1 model are obtained exactly from the conditional marginals of the true exponential model. A disease hypothesis is chosen randomly for the simulation with a probability proportional to the weights of the present diseases. The number of present diseases in the hypothesis is |**D**| = 2. All the marginal probabilities have been computed exactly for a small number of sign and disease variables (*N*_*S*_ = 20 and *N*_*D*_ = 5). The data are results of at least 500 independent realizations of the model and simulation process.

**Figure 6:**
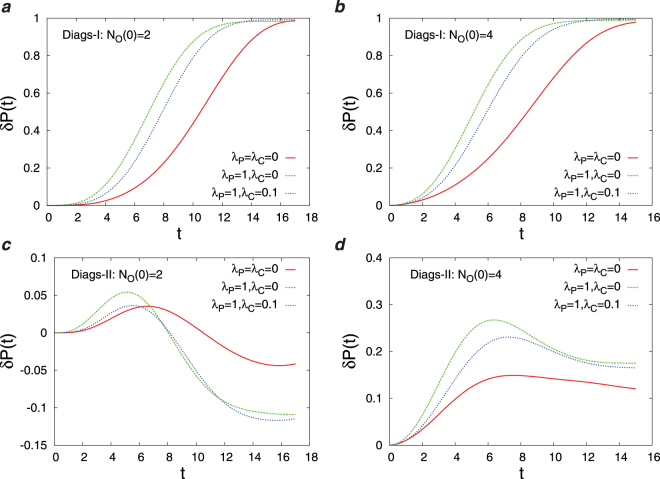
The difference *δP*(*t*) ≡ *P*(*T*_*R*_ ≤ *t*) − *P*(*T*_*W*_ ≤ *t*) in the cumulative probabilities of the first diagnosis times for different numbers of the initial observations *N*_*O*_(0) and the parameters *λ*_*P*_, *λ*_*C*_. The results have been obtained from the D2S1 model by (**a**),(**b**) the Diags-I (greedy), and (**c**)–(**d**) Diags-II (greedy) with the prior probabilities *P*_0_(*D*_*a*_ = 1) = 2/*N*_*D*_, and the threshold probability *P*_*th*_ = 0.9. The model parameters of the (fully connected) D2S1 model are obtained exactly from the conditional marginals of the true exponential model. A disease hypothesis is chosen randomly for the simulation with a probability proportional to the weights of the present diseases. The number of present diseases in the hypothesis is |**D**| = 2. All the marginal probabilities have been computed exactly for a small number of sign and disease variables (*N*_*S*_ = 20 and *N*_*D*_ = 5). The data are results of at least 500 independent realizations of the model and simulation process.

**Figure 7:**
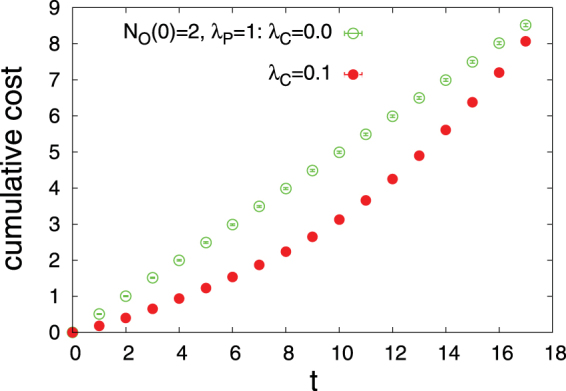
Cumulative cost of the diagnosis for two different values of *λ*_*C*_. The results have been obtained from the D2S1 model by the Diags-II (greedy) with the prior probabilities *P*_0_(*D*_*a*_ = 1) = 2/*N*_*D*_. The model parameters of the (fully connected) D2S1 model are obtained exactly from the conditional marginals of the true exponential model. A disease hypothesis is chosen randomly for the simulation with a probability proportional to the weights of the present diseases. All the marginal probabilities have been computed exactly for a small number of sign and disease variables (*N*_*S*_ = 20 and *N*_*D*_ = 5). The data are results of averaging over at least 500 independent realizations of the model and simulation process.

**Figure 8:**
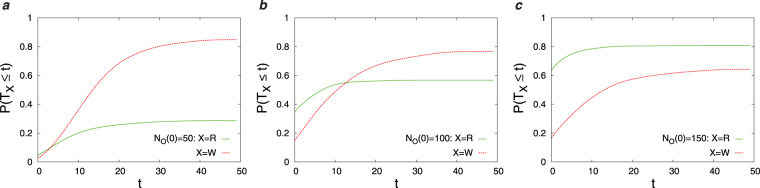
The cumulative probabilities *P*(*T*_*R*_ ≤ *t*) and *P*(*T*_*W*_ ≤ *t*) of the first diagnosis times for different numbers of the initial observations *N*_*O*_(0). The results have been obtained from a sparse D2S1 model by the Diags-II (random) with the prior probabilities *P*_0_(*D*_*a*_ = 1) = 2/*N*_*D*_, and the threshold probability *P*_*th*_ = 0.9. The model parameters of the (sparse) D2S1 model are obtained exactly from the conditional marginals of the true exponential model. The interaction graph and model parameters are: *N*_*S*_ = 500, *N*_*D*_ = 50, *M*_*a*_ = 50, *M*_*ab*_ = 100, *k*_*a*_ = 150, *k*_*ab*_ = 150. A disease hypothesis is chosen randomly for the simulation with a probability proportional to the weights of the present diseases. The number of present diseases in the hypothesis is |**D**| = 2. The marginal probabilities have been computed approximately by the Monte Carlo algorithm. The data are results of at least 200 independent realizations of the model and simulation process.

**Figure 9:**
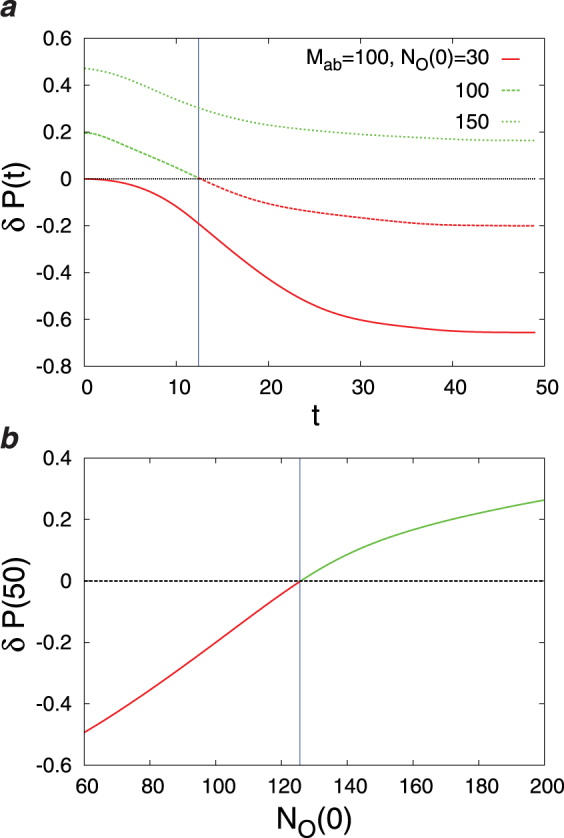
The difference *δP*(*t*) ≡ *P*(*T*_*R*_ ≤  *t*) − *P*(*T*_*W*_ ≤ *t*) in the cumulative probabilities of the first diagnosis times: (**a**) vs the number of observations *t* for different numbers of the initial observations *N*_*O*_(0), and (**b**) *δP*(50) vs *N*_*O*_(0) for a sufficiently large value of *t*. The results have been obtained from a sparse D2S1 model by the Diags-II (random) with the prior probabilities *P*_0_(*D*_*a*_ = 1) = 2/*N*_*D*_, and the threshold probability *P*_*th*_ = 0.9. The model parameters of the (sparse) D2S1 model are obtained exactly from the conditional marginals of the true exponential model. The interaction graph and the model parameters are: *N*_*S*_ = 500, *N*_*D*_ = 50, *M*_*a*_ = 50, *M*_*ab*_ = 100, *k*_*a*_ = 150, *k*_*ab*_ = 150. A disease hypothesis is chosen randomly for the simulation with a probability proportional to the weights of the present diseases. The number of present diseases in the hypothesis is |**D**| = 2. The marginal probabilities have been computed approximately by the Monte Carlo algorithm. The data are results of at least 200 independent realizations of the model and simulation process.

**Figure 10:**
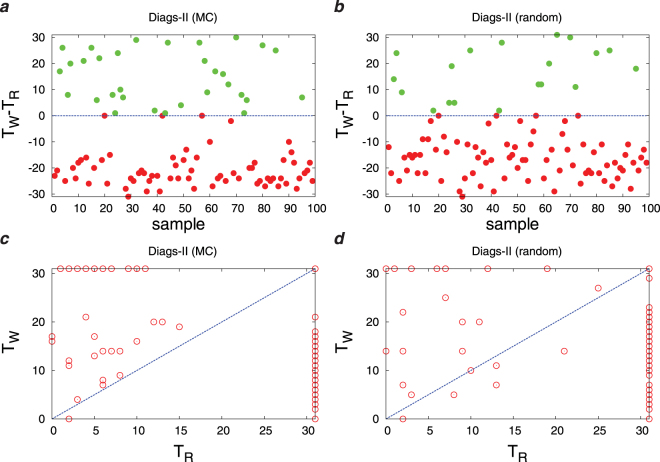
(**a**),(**b**) The difference *T*_*W*_ − *T*_*R*_ in the first diagnosis times, and (**c**),(**d**) *T*_*W*_ vs *T*_*R*_ for some independent realizations of the problem. The results have been obtained from a sparse D2S1 model by the Diags-II (zero-temperature MC) in panels (**a**)–(**c**), and the Diags-II (random) in panels (**b**)–(**d**). The prior probabilities are *P*_0_(*D*_*a*_ = 1) = 2/*N*_*D*_, and the threshold probability is *P*_*th*_ = 0.9. The model parameters of the (sparse) D2S1 model are obtained exactly from the conditional marginals of the true exponential model. The interaction graph and the model parameters are: *N*_*S*_ = 500, *N*_*D*_ = 50, *M*_*a*_ = 50, *M*_*ab*_ = 100, *k*_*a*_ = 150, *k*_*ab*_ = 150. The algorithms are given *N*_*O*_(0) = 50 initial observations to suggest a sequence of *T* = 30 other observations for diagnosis. Here we take *λ*_*P*_ = 1 and *λ*_*C*_ = 0. A disease hypothesis is chosen randomly for the simulation with a probability proportional to the weights of the present diseases. The number of present diseases in the hypothesis is |**D**| = 2. The marginal probabilities have been computed approximately by the Monte Carlo algorithm.

In this section, we present the results obtained by the numerical simulations of the Diags-I and Diags-II for different parameters in the objective function (*λ*_*P*_, *λ*_*C*_) and different number of initial observations *N*_*O*_(0). In Fig. 3 we report the overlap of the disease probabilities with the true disease values, *DL*(*t*), as the number of observations *t* increases starting from an initial number of observations. Here, we observe the impact of disease polarization and initial observations on *DL*(*t*) using the greedy strategy. Figure 4 displays the joint probability distribution of the first diagnosis times *P*(*T*_*R*_,*T*_*W*_) in the D2S1 model with the Diags-II (greedy). To see better the effects of (*λ*_*P*_, *λ*_*C*_) and *N*_*O*_(0) on the first diagnosis times, in Figs 5 and 6 we show the cumulative probability distributions *P*(*T*_*R*_ ≤ *t*) and *P*(*T*_*W*_ ≤ *t*). It is important to know how much the parameter *λ*_*C*_ reduces the cost of observations *SC*(*t*). Figure 7 displays the cumulative cost ${\sum }_{t^{\prime} =1}^{t}SC(t^{\prime} )$ for two values of *λ*_*C*_ we used in the numerical simulations. The number of variables in these figures is sufficiently small (*N*_*S*_ = 20, *N*_*D*_ = 5), which allows us to compute exactly the marginal sign/disease probabilities by an exhaustive sampling algorithm. To check the results for larger problem sizes, we have to resort to the Monte Carlo algorithms introduced in the previous sections. Figures 8–10 show the behavior of the first diagnosis times for the D2S1 model using the Diags-II. Here, we compare the results obtained by a random strategy with those that are obtained by maximizing the objective function.

Main points of this study are:

- Figs 3–6 show that adding a measure of disease polarization to the standard objective function (the maximum value of the log-likelihood) improves the diagnostic performance.
- We find that the cost of observations can considerably be decreased without seriously affecting the diagnostic performance. As Figs 3–7 show, the overlap of the disease probabilities with the true disease values does not significantly change by considering a small penalty for the cost of observations in the objective function. This is the case specially for intermediate values of *t*, where the optimized cumulative cost displays the largest deviation from the unoptimized one.
- The diagnostic performance of the Diags-I process always increases with the number of observations, because any observation (even if suggested randomly) reveals the true value of a previously unobserved sign. However, we see in Figs 3 and 6 that the performance of the Diags-II depends critically on the initial number of the observed signs. In particular, for ${N}_{O}(0) < {N}_{O}^{\ast }$ the Diags-II process more likely results in a wrong diagnosis, with *P*(*T*_*R*_ ≤ *T*) − *P*(*T*_*W*_ ≤ *T*) < 0 for a sufficiently large number of observations *T*. On the other hand, the probability of a right diagnosis in the Diags-II process is greater than the wrong one for ${N}_{O}(0) > {N}_{O}^{\ast }$. We obtain an upper bound for this critical value of ${N}_{O}^{\ast }$ for a sparse model of sign and disease variables, with *N*_*D*_ = 50, *N*_*S*_ = 500, *M*_*a*_ = 50, *M*_*ab*_ = 100, *k*_*a*_ = *k*_*ab*_ = 150, see Figs 8 and 9. The upper bound is obtained by the random strategy where at each step we choose randomly and uniformly an unobserved sign for observation. In general, we expect that such a critical value to be proportional to the total number of signs in the model, and of course dependent on the model structure. Here, the marginal sign/disease probabilities are computed by a standard Monte Carlo algorithm.
  Moreover, even for ${N}_{O}(0) < {N}_{O}^{\ast }$, there exists a characteristic number of observations t^*^, where for *t* < t^*^ the probability of inferring the right diseases in the Diags-II is still larger than that of the wrong diagnosis (Fig. 9). The characteristic time t^*^ of course increases with the number of initial observations. In other words, t^*^ gives the maximum number of observation tests (in the Diags-II) we can choose randomly before missing the useful information provided by the initial observations.
- We use the marginal sign probabilities *P*(*S*_*j*_ = + 1|**S**^*o*^) to guide the updating step of a (zero-temperature) Monte Carlo algorithm for optimizing the objective functional of the observations. This algorithm was used to uncover a small number (one or two) of hidden diseases in sparsely interacting models of signs and diseases, by simulating the Digas-II process. Figure 10 compares the first diagnosis times (*T*_*R*_, *T*_*W*_) obtained by the above algorithm with the ones predicted by the random strategy. Here, the marginal sign/disease probabilities and the maximum of the log-likelihood in the objective function are computed by the standard Monte Carlo and Simulated Annealing algorithms.

## Discussions and Conclusions

In this article, using novel, simple and rigorous models, we studied the efficiency determinants of a diagnostic approach, i.e. choice of a sequence of steps that leads to a diagnosis. We assessed the tradeoff between the number of steps (tests) and the cost (financial cost and biological risk) involved. We compared the efficiency of a sequential step-by-step diagnostic approach (i.e. a medical test is ordered and then the next test is decided) with an approach that orders a batch of tests at once during a clinical session. We recommend a combination of the two approaches, i.e. starting with the step-by-step approach and then switching to the batch approach would be optimal. The timing of when to switch is then dependent on the collected mass of information. At a certain critical point, switching the strategy would allow for faster clinical management. Moreover, we defined and reflected on an inherent property of a test, termed as disease polarization, that needs to be considered in constructing an efficient diagnostic flowchart. Our model includes interactions between diseases (and signs) which are typically neglected in the literature, but are emerging as important ingredients in omics analyses of human physiology and diseases^32^.

Finally, it should be mentioned that the typical diagnostic problems may involve many differentials (e.g. a few hundreds or thousands of diseases and signs)^13^. Monte Carlo is a computationally extensive algorithm to deal with large-scale problems. However, it works well independent of the model structure, if provided with adequate time. In our previous work, we proposed an approximate algorithm that is based on the Bethe approximation, but it works well for very sparse interaction graphs^23^. In a recent work, we are going to use the mean-field approximation, which again works well in fully-connected interaction graphs (unpublished data). Of course, the algorithms that are based on Bethe and mean-field approximations are more efficient than Monte Carlo. But, as mentioned earlier, their performance is limited by the structure of the model.

## Method

### Computing the objective function

Here we consider only the *D*1*S*1 and *D*2*S*1 models, where we can exactly compute the partition function

14 $$Z({\bf{D}})=\prod _{i}(2\,\cosh \,[{K}_{i}^{0}+\sum _{a}{K}_{i}^{a}{D}_{a}+\sum _{a < b}{K}_{i}^{ab}{D}_{a}{D}_{b}]).$$

For these models, we can also exactly compute the model parameters given the probabilities *P*_*true*_(*S*_*i*_|nodisease), *P*_*true*_(*S*_*i*_|only *D*_*a*_), and *P*_*true*_(*S*_*i*_|only *D*_*a*_, *D*_*b*_),

15 $${K}_{i}^{0}=\frac{1}{2}\,\mathrm{ln}(\frac{{P}_{true}({S}_{i}=+\mathrm{1|}{\rm{nodisease}})}{{P}_{true}({S}_{i}=-\mathrm{1|}{\rm{nodisease}})}),$$

16 $${K}_{i}^{a}=\frac{1}{2}\,\mathrm{ln}(\frac{{P}_{true}({S}_{i}=+\mathrm{1|}{\rm{only}}\,{D}_{a})}{{P}_{true}({S}_{i}=-\mathrm{1|}{\rm{only}}\,{D}_{a})})-{K}_{i}^{0},$$

17 $${K}_{i}^{ab}=\frac{1}{2}\,\mathrm{ln}(\frac{{P}_{true}({S}_{i}=+\mathrm{1|}{\rm{only}}\,{D}_{a},{D}_{b})}{{P}_{true}({S}_{i}=-\mathrm{1|}{\rm{only}}\,{D}_{a},{D}_{b})})-{K}_{i}^{0}-{K}_{i}^{a}-{K}_{i}^{b}.$$

For a given subset **O** of observed signs with values **S**^*o*^, the disease probabilities are obtained from

18 $$P({D}_{a}=\mathrm{1|}{{\bf{S}}}^{o})=\frac{1}{{\mathscr{Z}}({\bf{D}}|{{\bf{S}}}^{o})}\sum _{{\bf{D}}}{D}_{a}{e}^{- {\mathcal H} ({\bf{D}}|{{\bf{S}}}^{o})},$$

where $- {\mathcal H} ({\bf{D}}|{{\bf{S}}}^{o})\equiv \,\mathrm{log}\, {\mathcal L} ({\bf{D}}|{{\bf{S}}}^{o})$ is the log-likelihood function

19 $$ {\mathcal H} ({\bf{D}}|{{\bf{S}}}^{o})=-\sum _{a}{K}_{a}^{0}{D}_{a}-\sum _{i\in {\bf{O}}}{S}_{i}^{o}{h}_{i}({\bf{D}})+\sum _{i\in {\bf{O}}}\mathrm{ln}(2\,\cosh \,{h}_{i}({\bf{D}})),$$

and ${\mathscr{Z}}({\bf{D}}|{{\bf{S}}}^{o})$ is a normalization constant,

20 $${\mathscr{Z}}({\bf{D}}|{{\bf{S}}}^{o})\equiv \sum _{{\bf{D}}}{e}^{- {\mathcal H} ({\bf{D}}|{{\bf{S}}}^{o})}.$$

For brevity, here we defined the local field experienced by sign *i* as

21 $${h}_{i}({\bf{D}})\equiv {K}_{i}^{0}+\sum _{a}{K}_{i}^{a}{D}_{a}+\sum _{a < b}{K}_{i}^{ab}{D}_{a}{D}_{b}.$$

It is easy to show that the marginal probability of an unobserved sign is given by:

22 $$P({S}_{i}=\mathrm{1|}{{\bf{S}}}^{o})=\frac{1}{{\mathscr{Z}}({\bf{D}}|{{\bf{S}}}^{o})}\sum _{{\bf{D}}}(\frac{1+\,\tanh \,{h}_{i}({\bf{D}})}{2}){e}^{- {\mathcal H} ({\bf{D}}|{{\bf{S}}}^{o})}.$$

Now, we can use the standard Monte Carlo algorithm with the energy function ${\mathcal H} ({\bf{D}}|{{\bf{S}}}^{o})$, to compute the marginal probabilities which are needed for the objective function. More precisely, we need to sample the disease configurations with a probability proportional to $\exp (-\beta  {\mathcal H} ({\bf{D}}|{{\bf{S}}}^{o}))$. Here the inverse temperature parameter is *β* = 1. In addition, we need to compute the maximum log-likelihood function^29^, ${{\rm{\max }}}_{{\bf{D}}}\,\mathrm{log}\, {\mathcal L} ({\bf{D}}|{{\bf{S}}}^{o})$. This can be obtained by slowly increasing the inverse temperature parameter *β* in the above Monte Carlo algorithm. Note that in this way we obtain approximate values for the marginal probabilities and the maximum log-likelihood. The quality of these approximations of course depends on the computation time we spend for equilibration of the system in the Monte Carlo algorithm and the annealing process.

In practice, for a sparse D2S1 model with *N*_*D*_ = 50 diseases, *N*_*S*_ = 500 signs, and graph parameters *M*_*a*_ = 50, *M*_*ab*_ = 100, *k*_*a*_ = *k*_*ab*_ = 150, we run the algorithm for ${{\mathscr{N}}}_{total}=20000$ iterations (Monte Carlo sweeps) with ${{\mathscr{N}}}_{eq}=2000$ iterations for equilibration of the system, and extract the sample configurations after any ${{\mathscr{N}}}_{sample}=20$ iterations. To compute the maximum log-likelihood with the annealing algorithm, we increase linearly the inverse temperature parameter *β* from 1 to 10 in ${{\mathscr{N}}}_{annealing}=5000$ iterations. Altogether, computing all the necessary marginal probabilities and the objective function for a given sequence of *T* = 20 observations with the above parameters takes about 20 minutes of CPU time in a standard computer.

## Electronic supplementary material

Supplementary information
