# Supplementary material for: Uncovering hidden disease patterns by simulating clinical diagnostic processes
Source: Sci Rep. 2018 Feb 5;8:2436. doi: 10.1038/s41598-018-20826-y (PMC5799257; doi:10.1038/s41598-018-20826-y)
Supplement: Supplementary file 1 — Supplementary information [file 41598_2018_20826_MOESM1_ESM.pdf]

Supplementary information for "Uncovering hidden disease patterns by simulating clinical diagnostic processes"

Abolfazl Ramezanpour[a], Alireza Mashaghi[a*],

[a]*Leiden Academic Centre for Drug Research, Faculty of Mathematics and Natural Sciences, Leiden University, Leiden, The Netherlands and*

∗ *a.mashaghi.tabari@lacdr.leidenuniv.nl*

(Dated: December 8, 2017)

Appendix A: The one-disease-one-sign (D1S1) model

In the D1S1 model, we have only the one-disease interaction factors with local sign interactions. The model is completely identified with the model parameters K_i^0 and K_i^a in the following disease factors

$$\phi_0(\mathbf{S}) = e^{\sum_i K_i^0 S_i}, \qquad \phi_a(\mathbf{S}|D_a) = e^{D_a \sum_i K_i^a S_i}, \tag{A1}$$

with

$$P(\mathbf{S}|\mathbf{D}) = \frac{1}{Z(\mathbf{D})}\phi_0(\mathbf{S}) \times \prod_a \phi_a(\mathbf{S}|D_a). \tag{A2}$$

The exact expression for the normalization function $Z(\mathbf{D})$ reads as follows,

$$Z(\mathbf{D}) = \prod_i \left(2\cosh[K_i^0 + \sum_a K_i^a D_a]\right). \tag{A3}$$

Then, given the true marginal probabilities $P_{true}(S_i|\text{nodisease})$ and $P_{true}(S_i|\text{only}D_a)$, one can exactly obtain the model parameters [1],

$$K_i^0 = \frac{1}{2}\ln\left(\frac{P_{true}(S_i = +1|\text{nodisease})}{P_{true}(S_i = -1|\text{nodisease})}\right), \tag{A4}$$

$$K_i^a = \frac{1}{2}\ln\left(\frac{P_{true}(S_i = +1|\text{only}D_a)}{P_{true}(S_i = -1|\text{only}D_a)}\right) - K_i^0. \tag{A5}$$

Figures 1, 2, and 3 display the results we obtain with the D1S1 model. We take a small number of sign/disease variables to compute the marginal probabilities exactly by an exhaustive sampling algorithm. Here, the prior probabilities $P_0(D_a)$ are chosen such that the expected number of present diseases is one, i.e., $N_D P_0(D_a = 1) = 1$. From Ref. [1] we know that the D1S1 model does not work well when the number of present diseases in the hypothesis $|\mathbf{D}|$ is greater than one.

Appendix B: Including the sign-sign interactions (the D2S2 model)

In this section, we briefly describe the results we obtain for small instances of the D2S2 model, including also the two-sign interactions in the disease factors ϕ_a and ϕ_{ab}. More

precisely, the disease interaction factors are now given by

$$\phi_0(\mathbf{S}) = e^{\sum_i K_i^0 S_i}, \tag{B1}$$

$$\phi_a(\mathbf{S}|D_a) = e^{D_a[\sum_i K_i^a S_i + \sum_{i<j} K_{ij}^a S_i S_j]}, \tag{B2}$$

$$\phi_{ab}(\mathbf{S}|D_a, D_b) = e^{D_a D_b[\sum_i K_i^{ab} S_i + \sum_{i<j} K_{ij}^{ab} S_i S_j]}. \tag{B3}$$

Here, we can not exactly compute the partition function $Z(\mathbf{D})$, which appears in the conditional probability of the signs given the diseases, $P(\mathbf{S}|\mathbf{D})$. Suppose we are given the true marginal probabilities $P_{true}(S_i|\text{nodisease})$, $P_{true}(S_i, S_j|\text{only}D_a)$, and$P_{true}(S_i, S_j|\text{only}D_a, D_b)$. Then we use the Bethe approximation to estimate the model parameters $K_i^0, K_i^{a,ab}$, and $K_{ij}^{a,ab}$, as described in Ref. [1].

Note that there is no sign-sign interaction in the true exponential model used in the main text. Thus, for the true model here we take the following power-law distribution:

$$P_{true}(\mathbf{S}|\mathbf{D}) = \frac{1}{Z_{true}(\mathbf{D})} \frac{1}{(1 + H(\mathbf{S}, \mathbf{S}(\mathbf{D})))}. \tag{B4}$$

The Hamming distance $H(\mathbf{S}, \mathbf{S}') = \sum_i (S_i - S_i')^2/4$ gives the number of different signs in the two sign configurations. Here $\mathbf{S}(\mathbf{D})$ defines the signs attributed to $\mathbf{D}$. We will choose these signs randomly and uniformly from the configuration space of sign variables.

In Fig. 4, we compare the probabilities of the first diagnosis times $P(T_R \leq t)$ and $P(T_W \leq t)$ for the D2S2 model. Except for the model parameters which are estimated by the Bethe approximation, the other quantities are computed exactly by an exhaustive sampling algorithm. Here, we choose the prior probabilities $P_0(D_a)$ such that the expected number of disease probabilities which are greater than the threshold probability is about one. More precisely, the parameters K_a^0 are determined by $\sum_a \theta(P(D_a = 1) - P_{th}) \simeq 1$, where the Heaviside function $\theta(x)$ is one for $x > 0$, and zero otherwise.

---

[1] Ramezanpour, A., and Mashaghi, A. (2017). Toward First Principle Medical Diagnostics: On the Importance of Disease-Disease and Sign-Sign Interactions. Front. Phys. **5**:32.

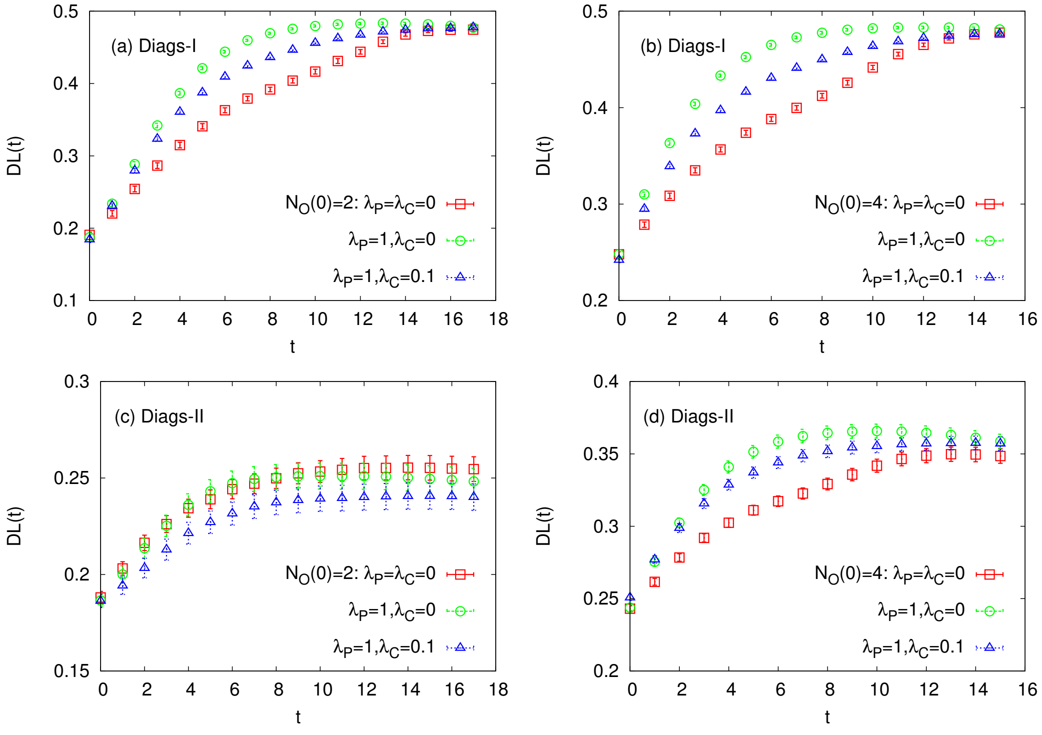

FIG. 1. Dependence of $DL(t)$ on the initial number of observed signs $N_O(0)$ and the parameters λ_P, λ_C. The results have been obtained from the D1S1 model by (a)-(b) the Diags-I (greedy), and (c)-(d) the Diags-II (greedy) with the prior probabilities $P_0(D_a = 1) = 1/N_D$. The model parameters of the (fully connected) D1S1 model are obtained exactly from the conditional marginals of the true exponential model. A disease hypothesis is chosen randomly for the simulation with a probability proportional to the weights of the present diseases. All the marginal probabilities have been computed exactly for a small number of sign and disease variables ($N_S = 20$ and $N_D = 5$). The data are results of averaging over at least 500 independent realizations of the model and simulation process.

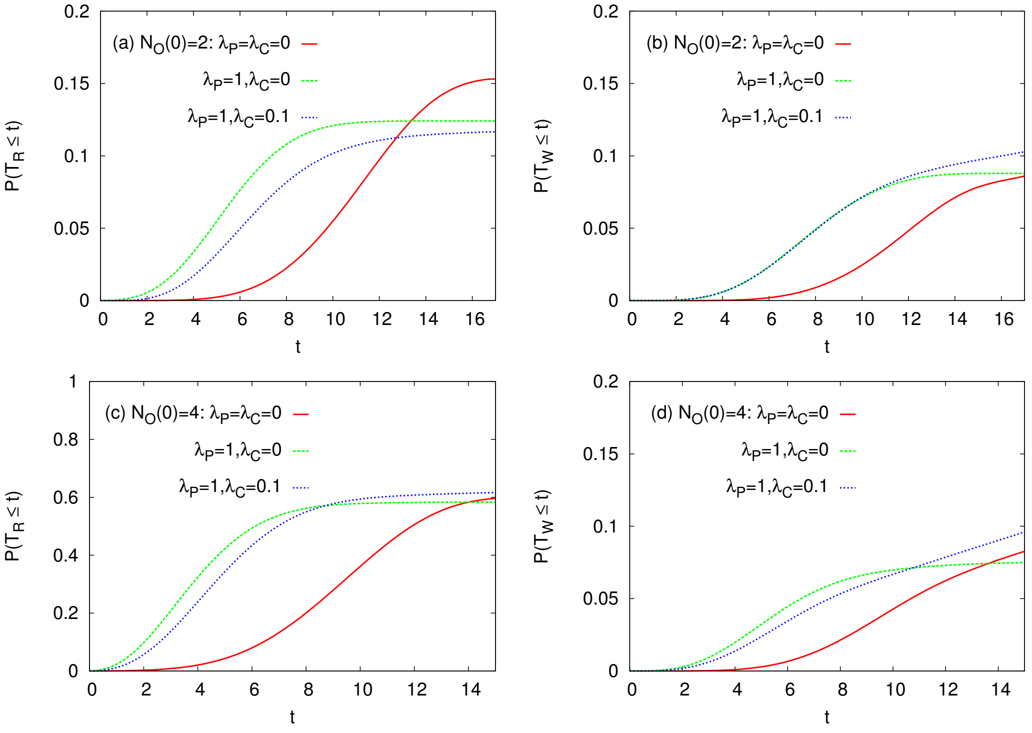

FIG. 2. The cumulative probabilities $P(T_R \leq t)$ and $P(T_W \leq t)$ of the first diagnosis times for different numbers of the initial observations $N_O(0)$ and the parameters λ_P, λ_C. The results have been obtained from the D1S1 model by the Diags-II (greedy) with the prior probabilities $P_0(D_a = 1) = 1/N_D$, and the threshold probability $P_{th} = 0.9$. The model parameters of the (fully connected) D1S1 model are obtained exactly from the conditional marginals of the true exponential model. A disease hypothesis is chosen randomly for the simulation with a probability proportional to the weights of the present diseases. The number of present diseases in the hypothesis is $|\mathbf{D}| = 1$. All the marginal probabilities have been computed exactly for a small number of sign and disease variables ($N_S = 20$ and $N_D = 5$). The data are results of at least 500 independent realizations of the model and simulation process.

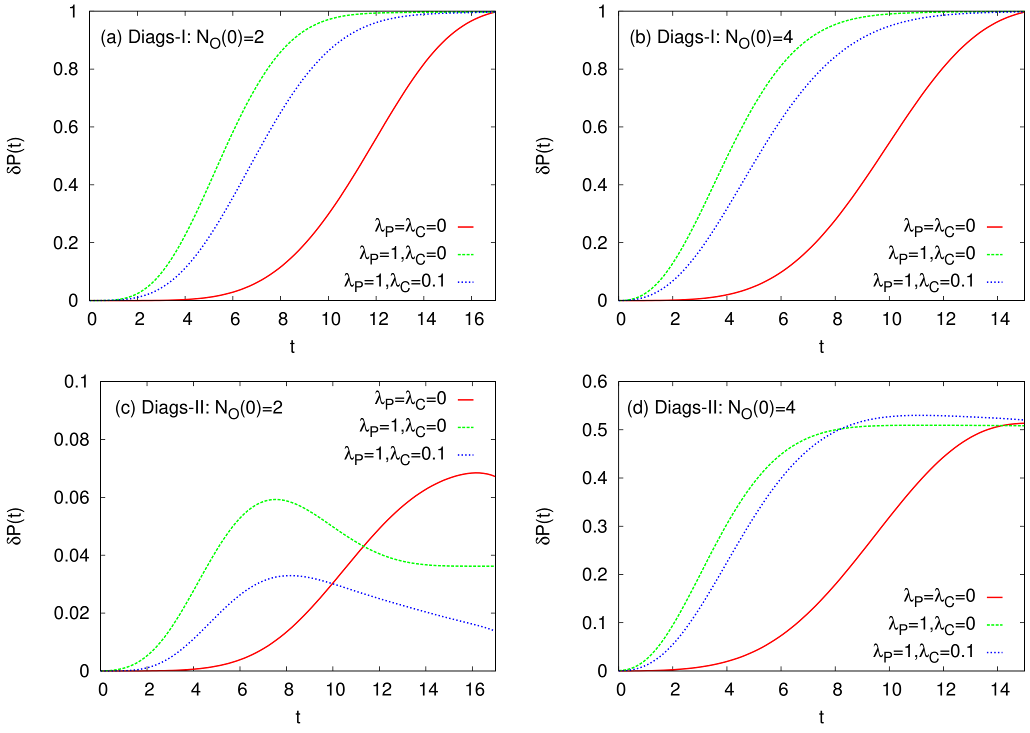

FIG. 3. The difference $\delta P(t) \equiv P(T_R \leq t) - P(T_W \leq t)$ in the cumulative probabilities of the first diagnosis times for different numbers of the initial observations $N_O(0)$ and the parameters λ_P, λ_C. The results have been obtained from the D1S1 model by (a)-(b) the Diags-I (greedy), and (c)-(d) Diags-II (greedy) with the prior probabilities $P_0(D_a = 1) = 1/N_D$, and the threshold probability $P_{th} = 0.9$. The model parameters of the (fully connected) D1S1 model are obtained exactly from the conditional marginals of the true exponential model. A disease hypothesis is chosen randomly for the simulation with a probability proportional to the weights of the present diseases. The number of present diseases in the hypothesis is $|\mathbf{D}| = 1$. All the marginal probabilities have been computed exactly for a small number of sign and disease variables ($N_S = 20$ and $N_D = 5$). The data are results of at least 500 independent realizations of the model and simulation process.

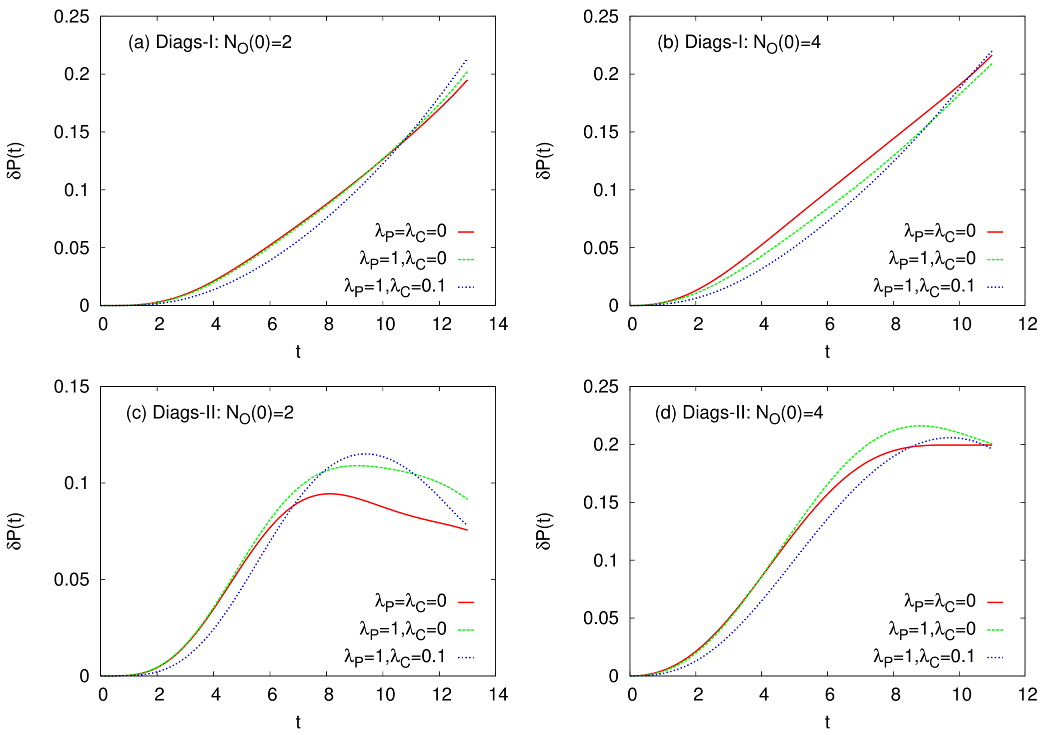

FIG. 4. The difference $\delta P(t) \equiv P(T_R \leq t) - P(T_W \leq t)$ in the cumulative probabilities of the first diagnosis times for different numbers of the initial observations $N_O(0)$ and the parameters λ_P, λ_C. The results have been obtained from the D2S2 model by (a)-(b) the Diags-I (greedy), and (c)-(d) Diags-II (greedy) with the prior probabilities $P_0(D_a = 1) = 0.7$, and the threshold probability $P_{th} = 0.9$. The model parameters of the (fully connected) D2S2 model are estimated approximately from the conditional marginals of the true power-law model. A disease hypothesis is chosen randomly for the simulation with a probability proportional to the weights of the present diseases. The number of present diseases in the hypothesis is $|\mathbf{D}| = 2$. All the marginal probabilities have been computed exactly for a small number of sign and disease variables ($N_S = 16$ and $N_D = 4$). The data are results of at least 500 independent realizations of the model and simulation process.

Appendix C: Codes

```
// This code uses the greedy strategy for a small number of sings
(NS) and diseases (ND)
// It works with fully-connected D1S1 or D2S1 models using Diags-I
or Diags-II
// It computes exactly the marginal probabilities to returns: DP(t),
DL(t), ML(t), SC(t)
// For the true model we take the exponential model



#include <iostream>
#include <fstream>
#include <sstream>
#include <cstdlib>
#include <math.h>
#include <vector>
#include <time.h>
#include <string>
using namespace std;


#define pow2(n) (1 << (n))

#define bit_map(x, n) (((x) & (1 << (n))) != 0)

#define bx(x)         ((x) - (((x)>>1) & 0x77777777)  \
                           - (((x)>>2) & 0x33333333)  \
                           - (((x)>>3) & 0x11111111))
#define count_bits(x)  (((bx(x)+(bx(x)>>4)) & 0x0F0F0F0F) % 255)

#define hdist(x, y) (count_bits((x)^(y)))


////////////////////// functions


void alloc_mem();

void make_PDS();

void make_WC();
void make_K();
void make_So();

void compute_E();
void compute_PDS();

void report(int sample);

double rang(int& seed);



////////////////////// end functions
```

```
////////////////////// variables


const int  ND=4;                // numbe of diseases
const int  NS=16;               // numbe of signs
const int  NO=2;                // number of initial observations
const int  M=1;                 // number of present diseases
const int  T=NS-NO;             // maximum length of the sequence of
observations
const int  Dmax=(1 << ND);      // number of disease patterns
const int  Smax=(1 << NS);      // number of sign patterns

const double  lambda_m=1.0;     // lagrange multiplier for maximum
likelihood
const double  lambda_p=1.0;     // lagrange multiplier for disease
polarization
const double  lambda_c=0.1;     // lagrange multiplier for sign cost

int seed;
int Nav;


double W;
double ML,DP,DC,DL;

vector<int>  SD;
vector<short int>  So;
vector<short int>  x,y;
vector<short int>  D,xd,yd;

vector<double>   weight,cost;

vector<double>   PS0,PD0,Px,Py;
vector<vector<double> >   PDS;

vector<double>   Ki,Ka;
vector<vector<double> >   Kia;
vector<vector<double> >   Pia;
vector<vector<vector<double> > >   Kiab;
vector<vector<vector<double> > >   Piab;

vector<double>   Et,avEt,eEt;
vector<double>   MLt,avMLt,eMLt;
vector<double>   DLt,avDLt,eDLt;
vector<double>   DPt,avDPt,eDPt;
vector<double>   DCt,avDCt,eDCt;
vector<double>   CCt,avCCt,eCCt;

vector<vector<double> >   PD,PS;
```

```
////////////////////// end variables





////////////////////// main

int main()
{

int to;
int imin;
double cmin;
double E,Emin;


seed=time(NULL);

alloc_mem();

for(int t=0;t<T;t++){
  avEt[t]=0;
  avMLt[t]=0;
  avDPt[t]=0;
  avDCt[t]=0;
  avCCt[t]=0;
  avDLt[t]=0;

  eEt[t]=0;
  eMLt[t]=0;
  eDPt[t]=0;
  eDCt[t]=0;
  eCCt[t]=0;
  eDLt[t]=0;
}

Nav=2000;
for(int sample=0;sample<Nav;sample++){          // averaging process

///

make_PDS();

make_K();

make_WC();

make_So();

///
```

```
to=0;
cmin=0;
CCt[0]=0;
while(to<T){                                   // simulation
process

  DCt[to]=cmin;

  compute_PDS();

  for(int a=0;a<ND;a++)PD[to][a]=Px[a];
  for(int i=0;i<NS;i++)PS[to][i]=Py[i];

  compute_E();

  ML=ML/(NO+to);

  MLt[to]=ML;
  DPt[to]=DP;
  DLt[to]=DL;
  Et[to]=-lambda_m*ML-lambda_p*DP+lambda_c*DCt[to];


  ///

  Emin=1e+6;
  for(int i0=0;i0<NS;i0++){            // the greedy step (choosing
the best unobserved sign)
  if(So[i0]==0){

  So[i0]=-1;
  if(PS[to][i0]>0.5)So[i0]=+1;

  compute_PDS();
  compute_E();

  ML=ML/(NO+to);
  E=-lambda_m*ML-lambda_p*DP+lambda_c*cost[i0];

  if(E<Emin){
    Emin=E;
    imin=i0;
  }
  So[i0]=0;

  }
  }
  cmin=cost[imin];
  if(to<T-1)CCt[to+1]=CCt[to]+cmin;

  So[imin]=-1;
  if(PS[to][imin]>0.5)So[imin]=+1;          // choose the most
probable value
  //So[imin]=yd[imin];                      // or the ture value
```

```
  ///

  to=to+1;

}

///


cout<<sample<<endl;

for(int t=0;t<T;t++){
  avEt[t]=avEt[t]+Et[t];
  avMLt[t]=avMLt[t]+MLt[t];
  avDPt[t]=avDPt[t]+DPt[t];
  avDCt[t]=avDCt[t]+DCt[t];
  avCCt[t]=avCCt[t]+CCt[t];
  avDLt[t]=avDLt[t]+DLt[t];

  eEt[t]=eEt[t]+Et[t]*Et[t];
  eMLt[t]=eMLt[t]+MLt[t]*MLt[t];
  eDPt[t]=eDPt[t]+DPt[t]*DPt[t];
  eDCt[t]=eDCt[t]+DCt[t]*DCt[t];
  eCCt[t]=eCCt[t]+CCt[t]*CCt[t];
  eDLt[t]=eDLt[t]+DLt[t]*DLt[t];
}
report(sample);

}


 return 0;
}
////////////////////// end  main




/////////////////// alloc_mem

void alloc_mem()
{


SD.resize(Dmax);

PDS.resize(Dmax);
for(int d=0;d<Dmax;d++){
PDS[d].resize(Smax);
}

D.resize(ND);
```

```
x.resize(ND);
y.resize(NS);

xd.resize(ND);
yd.resize(NS);

PD0.resize(ND);
PS0.resize(NS);

So.resize(NS);


///


Px.resize(ND);
Py.resize(NS);

PD.resize(T);
PS.resize(T);
for(int t=0;t<T;t++){
PD[t].resize(ND);
PS[t].resize(NS);
}

Et.resize(T);
MLt.resize(T);
DLt.resize(T);
DPt.resize(T);
DCt.resize(T);
CCt.resize(T);

avEt.resize(T);
avMLt.resize(T);
avDLt.resize(T);
avDPt.resize(T);
avDCt.resize(T);
avCCt.resize(T);

eEt.resize(T);
eMLt.resize(T);
eDLt.resize(T);
eDPt.resize(T);
eDCt.resize(T);
eCCt.resize(T);

///

Ki.resize(NS);
Ka.resize(ND);

Kia.resize(ND);
for(int a=0;a<ND;a++){
Kia[a].resize(NS);
```

```
}

Pia.resize(ND);
for(int a=0;a<ND;a++){
Pia[a].resize(NS);
}

Kiab.resize(ND);
for(int a=0;a<ND;a++){
Kiab[a].resize(a+1);
for(int b=0;b<a;b++){
Kiab[a][b].resize(NS);
}
}

Piab.resize(ND);
for(int a=0;a<ND;a++){
Piab[a].resize(a+1);
for(int b=0;b<a;b++){
Piab[a][b].resize(NS);
}
}

///


cost.resize(NS);

weight.resize(ND);



}
/////////////////// end  alloc_mem




//////////////////// make_PDS

void make_PDS()
{

int a0,b0,s0,check;
double Z,r,prod,P[NS];


//seed=1234;

///

for(int a=0;a<ND;a++){
  PD0[a]=float(M)/ND;
}
```

```
///

for(int i=0;i<NS;i++){
  PS0[i]=0.0;
}


///

Z=0;
s0=0;
SD[0]=s0;
for(int s=0;s<Smax;s++){
  r=exp(-hdist(s,s0));
  PDS[0][s]=r;
  Z=Z+r;
  for(int i=0;i<NS;i++){
    y[i]=bit_map(s,i);
    if(y[i]==1)PS0[i]=PS0[i]+r;
  }
}
for(int s=0;s<Smax;s++)PDS[0][s]=PDS[0][s]/Z;
for(int i=0;i<NS;i++)PS0[i]=PS0[i]/Z;

///

for(int d=1;d<Dmax;d++){

  check=0;
  for(int a=0;a<ND;a++){
    x[a]=bit_map(d,a);
    check=check+x[a];
  }
  for(int i=0;i<NS;i++)P[i]=0;


  Z=0;
  s0=1+rang(seed)*(Smax-1);
  SD[d]=s0;
  for(int s=0;s<Smax;s++){
    r=exp(-hdist(s,s0));
    PDS[d][s]=r;
    Z=Z+r;
    for(int i=0;i<NS;i++){
      y[i]=bit_map(s,i);
      if(y[i]==1)P[i]=P[i]+r;
    }
  }
  for(int s=0;s<Smax;s++)PDS[d][s]=PDS[d][s]/Z;

  if(check==1){
    for(int a=0;a<ND;a++){
      if(x[a]==1)a0=a;
```

```
    }
    for(int i=0;i<NS;i++){
      Pia[a0][i]=P[i]/Z;
    }
  }

  if(check==2){
    check=0;
    for(int a=0;a<ND;a++){
      if((x[a]==1)&&(check==0)){
        b0=a;
        check=1;
      }
      if((x[a]==1)&&(check==1))a0=a;
    }
    for(int i=0;i<NS;i++){
      Piab[a0][b0][i]=P[i]/Z;
    }
  }


}

///



}
////////////////////// end make_PDS




////////////////////// make_K

void make_K()
{



for(int i=0;i<NS;i++){
  Ki[i]=0.5*log(PS0[i]/(1.0-PS0[i]));
  //cout<<i<<" "<<Ki[i]<<endl;
}

///

for(int a=0;a<ND;a++){
    Ka[a]=log(PD0[a]/(1.0-PD0[a]));
  for(int i=0;i<NS;i++){
    Kia[a][i]=0.5*log(Pia[a][i]/(1.0-Pia[a][i]))-Ki[i];
    //cout<<a<<" "<<i<<" "<<Kia[a][i]<<endl;
  }
}
```

```
///

for(int a=0;a<ND;a++){
  for(int b=0;b<a;b++){
    for(int i=0;i<NS;i++){
      //Kiab[a][b][i]=0;
      Kiab[a][b][i]=0.5*log(Piab[a][b][i]/(1.0-Piab[a][b][i]))-
Ki[i]-Kia[a][i]-Kia[b][i];
      //cout<<a<<" "<<b<<" "<<i<<" "<<Kiab[a][b][i]<<endl;
    }
  }
}




}
////////////////////// end make_K





////////////////////// make_WC

void make_WC()
{

//seed=1234;

///

W=0;
for(int a=0;a<ND;a++){
  weight[a]=1.0*rang(seed);
  W=W+weight[a];
}

///

for(int i=0;i<NS;i++){
  cost[i]=rang(seed);
}

///




}
////////////////////// end make_WC
```

```
////////////////////// make_So

void make_So()
{

int nl,il;
int d0,s0,nd;
int N0,list[NS];
double rd,wd;
short int check;


check=0;
while(check==0){
d0=rang(seed)*Dmax;
nd=0;
rd=0;
wd=0;
for(int a=0;a<ND;a++){
  xd[a]=bit_map(d0,a);
  nd=nd+xd[a];
  rd=rd+xd[a]*rang(seed);
  wd=wd+xd[a]*weight[a];
}
if((nd==M)&&(rd<wd))check=1;
}
s0=SD[d0];

for(int i=0;i<NS;i++){
  So[i]=0;
  list[i]=i;
  yd[i]=2*bit_map(s0,i)-1;
}



N0=NS;
for(int l=0;l<NO;l++){
  nl=rang(seed)*N0;
  il=list[nl];
  N0=N0-1;
  list[nl]=list[N0];
  So[il]=yd[il];
}




}
////////////////////// end make_So
```

```
////////////////////////////////////////////////////////////////////
////////////////////////////////////////////////////////////////////
///




////////////////////// compute_PDS

void compute_PDS()
{

double Z,Zd,Zmax;
double summ0,summ1,summ2,prod;
short int check;


for(int a=0;a<ND;a++){
Px[a]=0;
}
for(int i=0;i<NS;i++){
Py[i]=0;
}

///

Z=0;
Zmax=-1;
for(int d=0;d<Dmax;d++){
for(int a=0;a<ND;a++)x[a]=bit_map(d,a);

///

summ0=0;
for(int a=0;a<ND;a++)summ0=summ0+x[a]*Ka[a];

summ1=summ0;
for(int i=0;i<NS;i++){
  if(So[i] != 0){
    summ2=Ki[i];
    for(int a=0;a<ND;a++){
      summ2=summ2+x[a]*Kia[a][i];
      for(int b=0;b<a;b++){
        summ2=summ2+x[a]*x[b]*Kiab[a][b][i];
      }
    }
    summ1=summ1+summ2*So[i]-log(2.0*cosh(summ2));
  }
}
Zd=exp(summ1);
```

```
if(Zd>Zmax){
  Zmax=Zd;
  for(int a=0;a<ND;a++)D[a]=x[a];
}

///

for(int s=0;s<Smax;s++){

check=1;
for(int i=0;i<NS;i++){
  y[i]=2*bit_map(s,i)-1;
  if(So[i]*y[i]<0)check=0;
}

if(check==1){

summ1=summ0;
for(int i=0;i<NS;i++){
    summ2=Ki[i];
    for(int a=0;a<ND;a++){
      summ2=summ2+x[a]*Kia[a][i];
      for(int b=0;b<a;b++){
        summ2=summ2+x[a]*x[b]*Kiab[a][b][i];
      }
    }
    summ1=summ1+summ2*y[i]-log(2.0*cosh(summ2));
}
prod=exp(summ1);
Z=Z+prod;

for(int a=0;a<ND;a++){
Px[a]=Px[a]+x[a]*prod;
}
for(int i=0;i<NS;i++){
Py[i]=Py[i]+(1+y[i])*prod/2;
}

}


}
}

///


for(int a=0;a<ND;a++){
Px[a]=Px[a]/Z;
}
for(int i=0;i<NS;i++){
Py[i]=Py[i]/Z;
}
```

```
}
////////////////////// end compute_PDS




////////////////////// compute_E

void compute_E()
{

  double summ1,summ2;


  summ1=0;
  summ2=0;
  for(int a=0;a<ND;a++){
    summ1=summ1+weight[a]*fabs(Px[a]-0.5);
    summ2=summ2+weight[a]*(Px[a]-0.5)*(2*xd[a]-1);
  }
  DP=summ1/W;
  DL=summ2/W;

  summ1=0;
  for(int a=0;a<ND;a++)summ1=summ1+Ka[a]*D[a];
  for(int i=0;i<NS;i++){
    if(So[i] != 0){
      summ2=Ki[i];
      for(int a=0;a<ND;a++){
          summ2 += Kia[a][i]*D[a];
          for(int b=0;b<a;b++){
            summ2 += Kiab[a][b][i]*D[a]*D[b];
          }
      }
      summ1 += So[i]*summ2-log(2*cosh(summ2));
    }
  }
  ML=summ1;



}
////////////////////// end compute_E





////////////////// report
void report(int sample)
{
```

```
double p1,p2;
double c1,c2;
double l1,l2;
double m1,m2;


ofstream output("avG.dat");
output<<"#samples="<<sample+1<<endl;
output<<"#t"<<" "<<"log-likelihood:ML(t),errors"<<"
"<<"polarization:DP(t),errors"<<" "<<"sign-cost:SC(t),errors"<<"
"<<"overlap-with-true-disease-pattern:DL(t),errors"<<endl;


for(int t=0;t<T;t++){

m1=avMLt[t]/(sample+1);
m2=eMLt[t]/(sample+1);
m2=sqrt((m2-m1*m1)/(sample+1));

p1=avDPt[t]/(sample+1);
p2=eDPt[t]/(sample+1);
p2=sqrt((p2-p1*p1)/(sample+1));

c1=avDCt[t]/(sample+1);
c2=eDCt[t]/(sample+1);
c2=sqrt((c2-c1*c1)/(sample+1));

l1=avDLt[t]/(sample+1);
l2=eDLt[t]/(sample+1);
l2=sqrt((l2-l1*l1)/(sample+1));

output<<t<<" "<<m1<<" "<<m2<<" "<<p1<<" "<<p2<<" "<<c1<<" "<<c2<<"
"<<l1<<" "<<l2<<endl;
}


}
/////////////////// report




////////////////////// rang

double rang(int& seed)
{

      int a, m, q, r, l;
      double conv, rand;


      a = 16807;
      m = 2147483647;
      q = 127773;
```

```
        r = 2836;

        conv = 1.0 / (m - 1);

        l = seed / q;
        seed = a * (seed - q * l) - r * l;
        if (seed < 0) {
                seed += m;
        }
        rand = conv * (seed - 1);



        return rand;
}
////////////////////// end rang
```

```
// This code uses the greedy strategy for a small number of sings
(NS) and diseases (ND)
// It works with the fully-connected D1S1 or D2S1 models using
Diags-I or Diags-II
// It computes exactly the marginal probabilities and returns:
P(T_R<t), P(T_W<t), and P(T_R,T_W)
// For the true model we take the exponential model


#include <iostream>
#include <fstream>
#include <sstream>
#include <cstdlib>
#include <math.h>
#include <vector>
#include <time.h>
#include <string>
using namespace std;


#define pow2(n) (1 << (n))

#define bit_map(x, n) (((x) & (1 << (n))) != 0)

#define bx(x)        ((x) - (((x)>>1) & 0x77777777)  \
                          - (((x)>>2) & 0x33333333)  \
                          - (((x)>>3) & 0x11111111))
#define count_bits(x)  (((bx(x)+(bx(x)>>4)) & 0x0F0F0F0F) % 255)

#define hdist(x, y) (count_bits((x)^(y)))


////////////////////// functions


void alloc_mem();

void make_PDS();

void make_WC();
void make_K();
void make_So();

void compute_E();
void compute_PDS();

void report(int sample);

double rang(int& seed);



////////////////////// end functions
```

```
////////////////////// variables


const int  ND=4;                // numbe of diseases
const int  NS=16;               // numbe of signs
const int  NO=2;                // number of initial observations
const int  M=1;                 // number of present diseases
const int  T=NS-NO;             // maximum length of the sequence of 
observations
const int  Dmax=(1 << ND);      // number of disease patterns
const int  Smax=(1 << NS);      // number of sign patterns

const double  Pmin=0.9;         // threshold value for accepting a 
disease
const double  lambda_m=1.0;     // lagrange multiplier for maximum 
likelihood
const double  lambda_p=1.0;     // lagrange multiplier for disease 
polarization
const double  lambda_c=0.1;     // lagrange multiplier for sign cost

int seed;                       // seed of random number generator
int Nav;                        // number of averaging
int TR,TW;                      // first right and wrong diagnosis 
times

short int check_r,check_w;

double W;
double ML,DP,DC,DL;

vector<int>  SD;
vector<short int>  So;
vector<short int>  x,y;
vector<short int>  D,xd,yd;

vector<vector<int> >   PT;

vector<double>   weight,cost;
vector<double>   FDTR,FDTW;

vector<double>   PS0,PD0,Px,Py;
vector<vector<double> >   PDS;

vector<double>   Ki,Ka;
vector<vector<double> >   Kia;
vector<vector<double> >   Pia;
vector<vector<vector<double> > >   Kiab;
vector<vector<vector<double> > >   Piab;

vector<vector<double> >   PD,PS;
```

```
////////////////////// end variables




////////////////////// main

int main()
{

int to;
int imin;
int cr,cw;
double E,Emin;


seed=time(NULL);

alloc_mem();

for(int t=0;t<T;t++){
    FDTR[t]=0;
    FDTW[t]=0;
}

for(int t1=0;t1<T+1;t1++){
  for(int t2=0;t2<T+1;t2++){
        PT[t1][t2]=0;
  }
}

Nav=2000;
for(int sample=0;sample<Nav;sample++){          // averaging process

///

make_PDS();

make_K();

make_WC();

make_So();

///

to=0;
cr=0;
cw=0;
TR=T;
TW=T;
```

```
check_r=0;
check_w=0;
while((to<T)&&(check_r+check_w <2)){            // simulation
process


  compute_PDS();

  for(int a=0;a<ND;a++)PD[to][a]=Px[a];
  for(int i=0;i<NS;i++)PS[to][i]=Py[i];

  ///

  check_r=0;
  check_w=0;
  for(int a=0;a<ND;a++){
    if((xd[a]==1)&&(PD[to][a]>Pmin))check_r=1;
    if((xd[a]==0)&&(PD[to][a]>Pmin))check_w=1;
  }


  if((check_r==1)&&(cr==0)){
    cr=1;
    TR=to;
    for(int t=to;t<T;t++)FDTR[t] += 1;
  }

  if((check_w==1)&&(cw==0)){
    cw=1;
    TW=to;
    for(int t=to;t<T;t++)FDTW[t] += 1;
  }

  if((check_r==0)||(check_w==0)){

  ///

  Emin=1e+6;
  for(int i0=0;i0<NS;i0++){             // the greedy step (choosing
the best unobserved sign)
  if(So[i0]==0){

  So[i0]=-1;
  if(PS[to][i0]>0.5)So[i0]=+1;

  compute_PDS();
  compute_E();

  ML=ML/(NO+to);
  E=-lambda_m*ML-lambda_p*DP+lambda_c*cost[i0];


  if(E<Emin){
    Emin=E;
```

```
    imin=i0;
  }
  So[i0]=0;

  }
  }

  ///

  So[imin]=-1;
  if(PS[to][imin]>0.5)So[imin]=+1;          // choose the most
probable value
  //So[imin]=yd[imin];                      // or the ture value

  }


  to=to+1;
}
PT[TR][TW] += 1;

///


cout<<sample<<" "<<TR<<" "<<TW<<endl;

report(sample);

}


 return 0;
}
////////////////////// end  main




//////////////////// alloc_mem

void alloc_mem()
{



SD.resize(Dmax);

PDS.resize(Dmax);
for(int d=0;d<Dmax;d++){
PDS[d].resize(Smax);
}

D.resize(ND);
```

```
x.resize(ND);
y.resize(NS);

xd.resize(ND);
yd.resize(NS);

PD0.resize(ND);
PS0.resize(NS);

So.resize(NS);


///


Px.resize(ND);
Py.resize(NS);

PD.resize(T);
PS.resize(T);
for(int t=0;t<T;t++){
PD[t].resize(ND);
PS[t].resize(NS);
}

PT.resize(T+1);
for(int t=0;t<T+1;t++){
PT[t].resize(T+1);
}

///

Ki.resize(NS);
Ka.resize(ND);

Kia.resize(ND);
for(int a=0;a<ND;a++){
Kia[a].resize(NS);
}

Pia.resize(ND);
for(int a=0;a<ND;a++){
Pia[a].resize(NS);
}

Kiab.resize(ND);
for(int a=0;a<ND;a++){
Kiab[a].resize(a+1);
for(int b=0;b<a;b++){
Kiab[a][b].resize(NS);
}
}

Piab.resize(ND);
```

```
for(int a=0;a<ND;a++){
Piab[a].resize(a+1);
for(int b=0;b<a;b++){
Piab[a][b].resize(NS);
}
}

///


cost.resize(NS);

weight.resize(ND);

FDTR.resize(T);
FDTW.resize(T);


}
/////////////////// end  alloc_mem




///////////////////// make_PDS

void make_PDS()
{

int a0,b0,s0,check;
double Z,r,prod,P[NS];


//seed=1234;

///

for(int a=0;a<ND;a++){
  PD0[a]=float(M)/ND;
  //PD0[a]=0.5;
}

///

for(int i=0;i<NS;i++){
  PS0[i]=0.0;
}


///

Z=0;
s0=0;
SD[0]=s0;
```

```
for(int s=0;s<Smax;s++){
  r=exp(-hdist(s,s0));
  PDS[0][s]=r;
  Z=Z+r;
  for(int i=0;i<NS;i++){
    y[i]=bit_map(s,i);
    if(y[i]==1)PS0[i]=PS0[i]+r;
  }
}
for(int s=0;s<Smax;s++)PDS[0][s]=PDS[0][s]/Z;
for(int i=0;i<NS;i++)PS0[i]=PS0[i]/Z;

///

for(int d=1;d<Dmax;d++){

  check=0;
  for(int a=0;a<ND;a++){
    x[a]=bit_map(d,a);
    check=check+x[a];
  }
  for(int i=0;i<NS;i++)P[i]=0;


  Z=0;
  s0=1+rang(seed)*(Smax-1);
  SD[d]=s0;
  for(int s=0;s<Smax;s++){
    r=exp(-hdist(s,s0));
    PDS[d][s]=r;
    Z=Z+r;
    for(int i=0;i<NS;i++){
      y[i]=bit_map(s,i);
      if(y[i]==1)P[i]=P[i]+r;
    }
  }
  for(int s=0;s<Smax;s++)PDS[d][s]=PDS[d][s]/Z;

  if(check==1){
    for(int a=0;a<ND;a++){
      if(x[a]==1)a0=a;
    }
    for(int i=0;i<NS;i++){
      Pia[a0][i]=P[i]/Z;
    }
  }

  if(check==2){
    check=0;
    for(int a=0;a<ND;a++){
      if((x[a]==1)&&(check==0)){
         b0=a;
         check=1;
      }
```

```
      if((x[a]==1)&&(check==1))a0=a;
    }
    for(int i=0;i<NS;i++){
      Piab[a0][b0][i]=P[i]/Z;
    }
  }


}

///



}
////////////////////// end make_PDS




////////////////////// make_K

void make_K()
{



for(int i=0;i<NS;i++){
  Ki[i]=0.5*log(PS0[i]/(1.0-PS0[i]));
  //cout<<i<<" "<<Ki[i]<<endl;
}

///

for(int a=0;a<ND;a++){
    Ka[a]=log(PD0[a]/(1.0-PD0[a]));
  for(int i=0;i<NS;i++){
    Kia[a][i]=0.5*log(Pia[a][i]/(1.0-Pia[a][i]))-Ki[i];
    //cout<<a<<" "<<i<<" "<<Kia[a][i]<<endl;
  }
}

///

for(int a=0;a<ND;a++){
  for(int b=0;b<a;b++){
    for(int i=0;i<NS;i++){
      Kiab[a][b][i]=0.0;
      //Kiab[a][b][i]=0.5*log(Piab[a][b][i]/(1.0-Piab[a][b][i]))-
Ki[i]-Kia[a][i]-Kia[b][i];
      //cout<<a<<" "<<b<<" "<<i<<" "<<Kiab[a][b][i]<<endl;
    }
  }
}
```

```
}
////////////////////// end make_K





////////////////////// make_WC

void make_WC()
{

//seed=1234;

///

W=0;
for(int a=0;a<ND;a++){
  weight[a]=rang(seed);
  W=W+weight[a];
}

///

for(int i=0;i<NS;i++){
  cost[i]=rang(seed);
}

///





}
////////////////////// end make_WC





////////////////////// make_So

void make_So()
{

int nl,il;
int d0,s0,nd;
int N0,list[NS];
double rd,wd;
short int check;
```

```
check=0;
while(check==0){
d0=rang(seed)*Dmax;
nd=0;
rd=0;
wd=0;
for(int a=0;a<ND;a++){
  xd[a]=bit_map(d0,a);
  nd=nd+xd[a];
  rd=rd+xd[a]*rang(seed);
  wd=wd+xd[a]*weight[a];
}
if((nd==M)&&(rd<wd))check=1;
}
s0=SD[d0];

for(int i=0;i<NS;i++){
  So[i]=0;
  list[i]=i;
  yd[i]=2*bit_map(s0,i)-1;
}



N0=NS;
for(int l=0;l<NO;l++){
  nl=rang(seed)*N0;
  il=list[nl];
  N0=N0-1;
  list[nl]=list[N0];
  So[il]=yd[il];
}




}
////////////////////// end make_So




//////////////////////////////////////////////////////////////////////
//////////////////////////////////////////////////////////////////////
///




////////////////////// compute_PDS

void compute_PDS()
```

```
{

double Z,Zd,Zmax;
double summ0,summ1,summ2,prod;
short int check;


for(int a=0;a<ND;a++){
Px[a]=0;
}
for(int i=0;i<NS;i++){
Py[i]=0;
}

///

Z=0;
Zmax=-1;
for(int d=0;d<Dmax;d++){
for(int a=0;a<ND;a++)x[a]=bit_map(d,a);

///
summ0=0;
for(int a=0;a<ND;a++)summ0=summ0+x[a]*Ka[a];

summ1=summ0;
for(int i=0;i<NS;i++){
  if(So[i] != 0){
    summ2=Ki[i];
    for(int a=0;a<ND;a++){
      summ2=summ2+x[a]*Kia[a][i];
      for(int b=0;b<a;b++){
        summ2=summ2+x[a]*x[b]*Kiab[a][b][i];
      }
    }
    summ1=summ1+summ2*So[i]-log(2.0*cosh(summ2));
  }
}
Zd=exp(summ1);

if(Zd>Zmax){
  Zmax=Zd;
  for(int a=0;a<ND;a++)D[a]=x[a];
}

///

for(int s=0;s<Smax;s++){

check=1;
for(int i=0;i<NS;i++){
  y[i]=2*bit_map(s,i)-1;
  if(So[i]*y[i]<0)check=0;
}
```

```
if(check==1){

summ1=summ0;
for(int i=0;i<NS;i++){
    summ2=Ki[i];
    for(int a=0;a<ND;a++){
      summ2=summ2+x[a]*Kia[a][i];
      for(int b=0;b<a;b++){
        summ2=summ2+x[a]*x[b]*Kiab[a][b][i];
      }
    }
    summ1=summ1+summ2*y[i]-log(2.0*cosh(summ2));
}
prod=exp(summ1);
Z=Z+prod;

for(int a=0;a<ND;a++){
Px[a]=Px[a]+x[a]*prod;
}
for(int i=0;i<NS;i++){
Py[i]=Py[i]+(1+y[i])*prod/2;
}

}


}
}

///


for(int a=0;a<ND;a++){
Px[a]=Px[a]/Z;
}
for(int i=0;i<NS;i++){
Py[i]=Py[i]/Z;
}



}
////////////////////// end compute_PDS






////////////////////// compute_E

void compute_E()
{
```

```
  double summ1,summ2;


  summ1=0;
  summ2=0;
  for(int a=0;a<ND;a++){
    summ1=summ1+weight[a]*fabs(Px[a]-0.5);
    summ2=summ2+weight[a]*(Px[a]-0.5)*(2*xd[a]-1);
  }
  DP=summ1/W;
  DL=summ2/W;

  summ1=0;
  for(int a=0;a<ND;a++)summ1=summ1+Ka[a]*D[a];
  for(int i=0;i<NS;i++){
    if(So[i] != 0){
      summ2=Ki[i];
      for(int a=0;a<ND;a++){
          summ2 += Kia[a][i]*D[a];
          for(int b=0;b<a;b++){
            summ2 += Kiab[a][b][i]*D[a]*D[b];
          }
      }
      summ1 += So[i]*summ2-log(2*cosh(summ2));
    }
  }
  ML=summ1;



}
////////////////////// end compute_E




/////////////////// report
void report(int sample)
{


ofstream output1("FDT.dat");
output1<<"#sampels="<<sample+1 <<endl;
output1<<"#t"<<" "<<"P(T_R)"<<" "<<"P(T_W)"<<" "<<"P(T_R)-
P(T_W)"<<endl;

for(int t=0;t<T;t++){
output1<<t<<" "<<FDTR[t]/(sample+1.0)<<" "<<FDTW[t]/(sample+1.0)<<"
"<<(FDTR[t]-FDTW[t])/(sample+1.0)<<endl;
}

///

ofstream output2("TRW.dat");
```

```
output2<<"#sampel="<<sample+1 <<endl;
output2<<"#T_R"<<" "<<"T_W"<<" "<<"P(T_R,T_W)"<<endl;

for(int t1=0;t1<T+1;t1++){
for(int t2=0;t2<T+1;t2++){
output2<<t1<<" "<<t2<<" "<<PT[t1][t2]/(sample+1.0)<<endl;
}
}


}
/////////////////// report




////////////////////// rang

double rang(int& seed)
{

      int a, m, q, r, l;
      double conv, rand;


      a = 16807;
      m = 2147483647;
      q = 127773;
      r = 2836;

      conv = 1.0 / (m - 1);

      l = seed / q;
      seed = a * (seed - q * l) - r * l;
      if (seed < 0) {
              seed += m;
      }
      rand = conv * (seed - 1);



      return rand;
}
////////////////////// end rang
```

```
// This code uses the zero-temperature Monte Carlo algorithm to find
an optimal sequence of T observations
// It works with sparse D1S1 or D2S1 models using Diags-II
// It computes approximately the marginal probabilities with the
standard Monte Carlo
// It computes approximately the maximum log-likelihood with a
Simulated Annealing algorithm
// It returns: DP(t), DL(t), ML(t), SC(t), T_R, T_W
// For the true model we take the exponential model


#include <iostream>
#include <fstream>
#include <sstream>
#include <cstdlib>
#include <math.h>
#include <vector>
#include <time.h>
#include <string>
using namespace std;


#define pow2(n) (1 << (n))

#define bit_map(x, n) (((x) & (1 << (n))) != 0)

#define bx(x)        ((x) - (((x)>>1) & 0x77777777)  \
                             - (((x)>>2) & 0x33333333)  \
                             - (((x)>>3) & 0x11111111))
#define count_bits(x)  (((bx(x)+(bx(x)>>4)) & 0x0F0F0F0F) % 255)

#define hdist(x, y) (count_bits((x)^(y)))


////////////////////// functions


void alloc_mem();

void make_G();

void make_WC();
void make_K();
void make_So();

void initial_seq();
void update_seq(int ti);

void make_O(int N);

double compute_ML();
double compute_hi(int i);
double compute_dL(int a);
```

```
void initial_D();
void compute_PDS();
void compute_Dmax();

void compute_E();

void report();

double rang(int& seed);



////////////////////// end functions





////////////////////// variables


const int  ND=20;                  // numbe of diseases (or one-
disease interaction factors)
const int  NS=200;                 // numbe of signs
const int  NC=50;                  // number of two-disease
interaction factors
const int  NO=20;                  // number of initial
observations
const int  da=100;                 // degree of one-disease
interaction factors
const int  dc=100;                 // degree of two-disease
interaction factors
const int  tmax=15;                // number of zero-temperature
Monte Carlo sweeps

const int  M=2;                    // number of present diseases
const int  T=10;                   // length of sequence of
observations

const double  Pth=0.9;             // threshold value for accepting
a disease
const double  lambda_m=1.0;        // lagrange multiplier for
maximum likelihood
const double  lambda_p=1.0;        // lagrange multiplier for
disease polarization
const double  lambda_c=0.1;        // lagrange multiplier for sign
cost

int seed;                          // seed of random number
generator
int TR,TW;                         // first right and wrong
diagnosis times

const int tmax_ML=4000;            // number of Monte Carlo sweeps
for computing maximum likelihood
```

```
const int tmax_EQ=2000;             // number of Monte Carlo sweeps
for equilibration
const int tmax_MC=20000;            // total number of Monte Carlo
sweeps

double W;
double ML,MLmax;
double DP,DC,MD;
double E,E_opt;

vector<int>  O;
vector<int>  seq,tim;
vector<int>  seq_new;
vector<vector<short int> >  SDa,SDc;

vector<short int>  So;
vector<short int>  D,Dmax;
vector<short int>  xd,yd;

vector<double>   weight,cost;

vector<double>   Px,Py;

vector<double>   Ki,Ka;
vector<vector<double> >   Kai,Kci;

vector<double>   DLt,DLt_new;
vector<double>   DPt,DPt_new;
vector<double>   DCt,DCt_new;
vector<double>   MDt,MDt_new;
vector<double>   DLt_0,DLt_0_new;
vector<double>   DLt_1,DLt_1_new;
vector<double>   DPt_0,DPt_0_new;
vector<double>   DPt_1,DPt_1_new;

vector<vector<double> >   PD,PS;
vector<vector<double> >   PDnew,PSnew;

vector<int>  kia,kic;
vector<vector<int> >  via,vai,vic,vci,vca;
vector<vector<int> >  vial,vail,vicl,vcil;


////////////////////// end variables






////////////////////// main

int main()
{
```

```
int ti;


alloc_mem();

seed=time(NULL);

make_G();

make_K();

make_WC();

make_So();

seed=time(NULL);

/////////////////////// zero-temprature Monte Carlo


ofstream output_E("E.dat");

///

initial_seq();

cout<< 0 <<"  "<< MD/T<<" "<< DP/T<<" "<< DC/T<<" "<< E/T<<" "<<
E_opt/T<<" "<< TR<<" "<< TW<<endl;
output_E<< 0 <<" "<< MD/T<<" "<< DP/T<<" "<< DC/T<<" "<< E/T<<" "<<
E_opt/T<<" "<< TR<<" "<< TW<<endl;

///

for(int t=1;t<tmax;t++){


  for(int lo=0;lo<T;lo++){
      ti=1+rang(seed)*T;
      update_seq(ti);
  }

  cout<< t <<"  "<< MD/T<<" "<< DP/T<<" "<< DC/T<<" "<< E/T<<" "<<
E_opt/T<<" "<< TR<<" "<< TW<<endl;
  output_E<< t <<"  "<< MD/T<<" "<< DP/T<<" "<< DC/T<<" "<< E/T<<"
"<< E_opt/T<<" "<< TR<<" "<< TW<<endl;

}

///


 return 0;
}
```

}

```
////////////////////// end  main




/////////////////// alloc_mem

void alloc_mem()
{

int kmax=max(ND,NC);


SDa.resize(ND);
for(int a=0;a<ND;a++){
SDa[a].resize(NS);
}

SDc.resize(NC);
for(int c=0;c<NC;c++){
SDc[c].resize(NS);
}

xd.resize(ND);
yd.resize(NS);

D.resize(ND);
Dmax.resize(ND);

O.resize(ND);

So.resize(NS);

tim.resize(NS);

seq.resize(T+1);
seq_new.resize(T+1);

///


Px.resize(ND);
Py.resize(NS);

PD.resize(T+1);
PS.resize(T+1);
for(int t=0;t<T+1;t++){
PD[t].resize(ND);
PS[t].resize(NS);
}

PDnew.resize(T+1);
PSnew.resize(T+1);
for(int t=0;t<T+1;t++){
```

```
PDnew[t].resize(ND);
PSnew[t].resize(NS);
}

DLt.resize(T+1);
DPt.resize(T+1);
DCt.resize(T+1);
MDt.resize(T+1);

DLt_new.resize(T+1);
DPt_new.resize(T+1);
DCt_new.resize(T+1);
MDt_new.resize(T+1);

DPt_0.resize(T+1);
DPt_1.resize(T+1);
DPt_0_new.resize(T+1);
DPt_1_new.resize(T+1);

DLt_0.resize(T+1);
DLt_1.resize(T+1);
DLt_0_new.resize(T+1);
DLt_1_new.resize(T+1);

///

Ki.resize(NS);
Ka.resize(ND);

Kai.resize(ND);
for(int a=0;a<ND;a++){
Kai[a].resize(da);
}

Kci.resize(NC);
for(int c=0;c<NC;c++){
Kci[c].resize(dc);
}

///

cost.resize(NS);

weight.resize(ND);

///


kia.resize(NS);
kic.resize(NS);

via.resize(NS);
vial.resize(NS);
for(int i=0;i<NS;i++){
```

```
via[i].resize(kmax);
vial[i].resize(kmax);
}

vai.resize(ND);
vail.resize(ND);
for(int a=0;a<ND;a++){
vai[a].resize(da);
vail[a].resize(da);
}

vic.resize(NS);
vicl.resize(NS);
for(int i=0;i<NS;i++){
vic[i].resize(kmax);
vicl[i].resize(kmax);
}

vci.resize(NC);
vcil.resize(NC);
for(int c=0;c<NC;c++){
vci[c].resize(dc);
vcil[c].resize(dc);
}

vca.resize(NC);
for(int c=0;c<NC;c++){
vca[c].resize(2);
}

///



}
/////////////////// end  alloc_mem




///////////////////// make_G

void make_G()
{

int j,a1,a2,d,li,lj,la;
short int check;
int ln,n,list[NS];


for(int i=0;i<NS;i++){
    kia[i]=0;
    kic[i]=0;
}
```

```
/////////

for(int a=0;a<ND;a++){

///

  for(int i=0;i<NS;i++){
      SDa[a][i]=-1;
      if(rang(seed)>0.5)SDa[a][i]=+1;
      list[i]=i;
  }

///

  n=NS;
  for(int la=0;la<da;la++){

    ln=rang(seed)*n;
    j=list[ln];
    n=n-1;
    list[ln]=list[n];

    lj=kia[j];
    vai[a][la]=j;
    via[j][lj]=a;
    vail[a][la]=lj;
    vial[j][lj]=la;
    kia[j]=lj+1;

  }

///

}


/////////


for(int c=0;c<NC;c++){

    check=0;
    while(check==0){
        check=1;
        a1=rang(seed)*ND;
        a2=rang(seed)*ND;
        if(a1==a2)check=0;
        for(int l=0;l<c;l++){
            if((vca[l][0]==a1)&&(vca[l][1]==a2))check=0;
            if((vca[l][0]==a2)&&(vca[l][1]==a1))check=0;
        }
    }
    vca[c][0]=a1;
```

```
    vca[c][1]=a2;

///

  for(int i=0;i<NS;i++){
      SDc[c][i]=-1;
      if(rang(seed)>0.5)SDc[c][i]=+1;
      list[i]=i;
  }

///

  n=NS;
  for(int lc=0;lc<dc;lc++){

    ln=rang(seed)*n;
    j=list[ln];
    n=n-1;
    list[ln]=list[n];

    lj=kic[j];
    vci[c][lc]=j;
    vic[j][lj]=c;
    vcil[c][lc]=lj;
    vicl[j][lj]=lc;
    kic[j]=lj+1;

  }

///

}



}
////////////////////// end make_G




////////////////////// make_K

void make_K()
{

int i,a,b;
int la,lb;
short int xa,xb;
double P;
double z=1+exp(-1.0);


for(int i=0;i<NS;i++){
```

```
  P=exp(-1.0)/z;
  Ki[i]=0.5*log(P/(1.0-P));

  //cout<<i<<" "<<Ki[i]<<endl;
}

///////


for(int a=0;a<ND;a++){

  P=float(M)/ND;
  Ka[a]=log(P)-log(1-P);

  for(int la=0;la<da;la++){
    i=vai[a][la];

    P=exp(-1.0)/z;
    if(SDa[a][i]==1)P=1.0/z;
    Kai[a][la]=0.5*log(P/(1.0-P))-Ki[i];
    //cout<<a<<" "<<i<<" "<<Kia[a][la]<<endl;
  }

}

///////


for(int c=0;c<NC;c++){
   for(int lc=0;lc<dc;lc++){
      i=vci[c][lc];
      a=vca[c][0];
      b=vca[c][1];

      la=0;
      lb=0;
      xa=0;
      xb=0;
      for(int l=0;l<da;l++){
          if(vai[a][l]==i){
              la=l;
              xa=1;
          }
          if(vai[b][l]==i){
              lb=l;
              xb=1;
          }
      }

      P=exp(-1.0)/z;
      if(SDc[c][i]==1)P=1.0/z;

      Kci[c][lc]=0.5*log(P/(1.0-P))-Ki[i]-xa*Kai[a][la]-xb*Kai[b]
```

```
[lb];

      //cout<<a<<" "<<b<<" "<<i<<" "<<Kic[c][lc]<<endl;

   }
}

////////


}
////////////////////// end make_K




////////////////////// make_WC

void make_WC()
{


W=0;
for(int a=0;a<ND;a++){
  weight[a]=1.0*rang(seed);
  W=W+weight[a];
}

///

for(int i=0;i<NS;i++){
  cost[i]=rang(seed);
}

///


}
////////////////////// end make_WC




////////////////////// make_So

void make_So()
{

int nl,il;
int c0,a0,b0;
int N0,list[NS];
short int check;
```

```
for(int a=0;a<ND;a++)xd[a]=0;

///


if(M==1){

  check=0;
  while(check==0){
    a0=rang(seed)*ND;
    if(rang(seed)<weight[a0])check=1;
  }
  xd[a0]=1;
  for(int i=0;i<NS;i++)yd[i]=SDa[a0][i];


}else if(M==2){

  check=0;
  while(check==0){
    c0=rang(seed)*NC;
    a0=vca[c0][0];
    b0=vca[c0][1];
    if(rang(seed)+rang(seed)<weight[a0]+weight[b0])check=1;
  }
  xd[a0]=1;
  xd[b0]=1;
  for(int i=0;i<NS;i++)yd[i]=SDc[c0][i];

}

///

for(int i=0;i<NS;i++){
  So[i]=0;
  list[i]=i;
  tim[i]=NS;
}

N0=NS;
for(int l=0;l<NO;l++){
  nl=rang(seed)*N0;
  il=list[nl];
  N0=N0-1;
  list[nl]=list[N0];
  So[il]=yd[il];
  tim[il]=0;
}
```

```
}
////////////////////// end make_So




////////////////////// make_O

void make_O(int N)
{

int l;
int n;
int t;
int list[N];


n=N;
for(int i=0;i<N;i++)list[i]=i;

t=0;
while(n>0){
l=rang(seed)*n;
O[t]=list[l];
n=n-1;
list[l]=list[n];
t=t+1;
}


}
////////////////////// end make_O




////////////////////////////////////////////////////////////////
SA ////////////////////////////////////////////////////////////////
//////////




////////////////////// initial_seq

void initial_seq()
{

int to;
int imax;
int cr,cw;
int N0,nl,list[NS];
double summ1,summ2;
```

```
double Pmax;
double W0,W1,P0,P1,L0,L1;

cr=0;
cw=0;
TR=T+1;
TW=T+1;

////////////

initial_D();
compute_PDS();
compute_Dmax();


for(int a=0;a<ND;a++)PD[0][a]=Px[a];
for(int i=0;i<NS;i++)PS[0][i]=Py[i];

P0=0;
P1=0;
L0=0;
L1=0;
W0=0;
W1=0;
summ1=0;
summ2=0;
for(int a=0;a<ND;a++){
    summ1=summ1+weight[a]*fabs(Px[a]-0.5);
    summ2=summ2+weight[a]*(Px[a]-0.5)*(2*xd[a]-1);
    if(xd[a]==0){
        W0=W0+weight[a];
        P0=P0+weight[a]*fabs(Px[a]-0.5);
        L0=L0+weight[a]*(Px[a]-0.5)*(2*xd[a]-1);
    }else{
        W1=W1+weight[a];
        P1=P1+weight[a]*fabs(Px[a]-0.5);
        L1=L1+weight[a]*(Px[a]-0.5)*(2*xd[a]-1);
    }
    if((xd[a]==1)&&(Px[a]>Pth)&&(cr==0)){
        cr=1;
        TR=0;
    }
    if((xd[a]==0)&&(Px[a]>Pth)&&(cw==0)){
        cw=1;
        TW=0;
    }
}
DPt[0]=summ1/W;
DLt[0]=summ2/W;
DPt_0[0]=P0/W0;
DPt_1[0]=P1/W1;
DLt_0[0]=L0/W0;
DLt_1[0]=L1/W1;
MDt[0]=MLmax/NO;
```

```
DCt[0]=0;
seq[0]=-1;

MDt_new[0]=MDt[0];
DPt_new[0]=DPt[0];
DCt_new[0]=DCt[0];
DLt_new[0]=DLt[0];
DPt_0_new[0]=DPt_0[0];
DPt_1_new[0]=DPt_1[0];
DLt_0_new[0]=DLt_0[0];
DLt_1_new[0]=DLt_1[0];
for(int a=0;a<ND;a++)PDnew[0][a]=PD[0][a];
for(int i=0;i<NS;i++)PSnew[0][i]=PS[0][i];

////////////

N0=0;
for(int i=0;i<NS;i++){
    if(So[i]==0){
        list[N0]=i;
        N0=N0+1;
    }
}

////////////

DP=0;
DC=0;
MD=0;
to=1;
while(to<=T){

  nl=rang(seed)*N0;
  imax=list[nl];
  N0=N0-1;
  list[nl]=list[N0];

  So[imax]=-1;
  if(PS[to-1][imax]>0.5)So[imax]=+1;
  tim[imax]=to;
  DCt[to]=cost[imax];
  seq[to]=imax;

  ///

  initial_D();
  compute_PDS();
  compute_Dmax();

  for(int a=0;a<ND;a++)PD[to][a]=Px[a];
  for(int i=0;i<NS;i++)PS[to][i]=Py[i];

  P0=0;
  P1=0;
```

```
  L0=0;
  L1=0;
  W0=0;
  W1=0;
  summ1=0;
  summ2=0;
  for(int a=0;a<ND;a++){
    summ1=summ1+weight[a]*fabs(Px[a]-0.5);
    summ2=summ2+weight[a]*(Px[a]-0.5)*(2*xd[a]-1);
    if(xd[a]==0){
        W0=W0+weight[a];
        P0=P0+weight[a]*fabs(Px[a]-0.5);
        L0=L0+weight[a]*(Px[a]-0.5)*(2*xd[a]-1);
    }else{
        W1=W1+weight[a];
        P1=P1+weight[a]*fabs(Px[a]-0.5);
        L1=L1+weight[a]*(Px[a]-0.5)*(2*xd[a]-1);
    }
    if((xd[a]==1)&&(Px[a]>Pth)&&(cr==0)){
        cr=1;
        TR=to;
    }
    if((xd[a]==0)&&(Px[a]>Pth)&&(cw==0)){
        cw=1;
        TW=to;
    }
  }
  DPt[to]=summ1/W;
  DLt[to]=summ2/W;
  DPt_0[to]=P0/W0;
  DPt_1[to]=P1/W1;
  DLt_0[to]=L0/W0;
  DLt_1[to]=L1/W1;
  MDt[to]=MLmax/(NO+to);

  DP=DP+DPt[to];
  DC=DC+DCt[to];
  MD=MD+MDt[to];


  ///

  to=to+1;

}
E=-lambda_m*MD-lambda_p*DP+lambda_c*DC;

E_opt=E;
report();


}
////////////////////// end initial_seq
```

```
/////////////////// update_seq
void update_seq(int ti)
{

int i0,l0,s0,N0;
int list[NS];
int TRnew,TWnew;
short int cr,cw;
short int check;
short int Sold[NS];
double summ1,summ2;
double MDnew,DPnew,DCnew;
double W0,W1,P0,P1,L0,L1;


cr=0;
cw=0;
TRnew=T+1;
TWnew=T+1;
if(TR<ti){
    cr=1;
    TRnew=TR;
}
if(TW<ti){
    cw=1;
    TWnew=TW;
}

//////////

MDnew=0;
DPnew=0;
DCnew=0;
for(int t=1;t<ti;t++){
  MDnew=MDnew+MDt[t];
  DPnew=DPnew+DPt[t];
  DCnew=DCnew+DCt[t];
}
for(int i=0;i<NS;i++)PSnew[ti-1][i]=PS[ti-1][i];

///

N0=0;
for(int i=0;i<NS;i++){
  Sold[i]=So[i];
  if(tim[i]>=ti){
      So[i]=0;
      list[N0]=i;
      N0=N0+1;
  }
```

```
}

///

for(int t=ti;t<T+1;t++){

  check=0;
  while(check==0){
    l0=rang(seed)*N0;
    i0=list[l0];
    if(rang(seed)<PSnew[t-1][i0])check=1;
  }
  N0=N0-1;
  list[l0]=list[N0];

  seq_new[t]=i0;

  s0=-1;
  if(PSnew[t-1][i0]>0.5)s0=+1;
  So[i0]=s0;

  DCnew=DCnew+cost[i0];
  DCt_new[t]=cost[i0];

  initial_D();
  compute_PDS();
  compute_Dmax();

  P0=0;
  P1=0;
  L0=0;
  L1=0;
  W0=0;
  W1=0;
  summ1=0;
  summ2=0;
  for(int a=0;a<ND;a++){
    PDnew[t][a]=Px[a];
    summ1=summ1+weight[a]*fabs(Px[a]-0.5);
    summ2=summ2+weight[a]*(Px[a]-0.5)*(2*xd[a]-1);
    if(xd[a]==0){
        W0=W0+weight[a];
        P0=P0+weight[a]*fabs(Px[a]-0.5);
        L0=L0+weight[a]*(Px[a]-0.5)*(2*xd[a]-1);
    }else{
        W1=W1+weight[a];
        P1=P1+weight[a]*fabs(Px[a]-0.5);
        L1=L1+weight[a]*(Px[a]-0.5)*(2*xd[a]-1);
    }
    if((xd[a]==1)&&(Px[a]>Pth)&&(cr==0)){
        cr=1;
        TRnew=t;
    }
    if((xd[a]==0)&&(Px[a]>Pth)&&(cw==0)){
```

```
        cw=1;
        TWnew=t;
      }
    }
    summ1=summ1/W;
    summ2=summ2/W;
    DPnew=DPnew+summ1;
    DPt_new[t]=summ1;
    DLt_new[t]=summ2;
    DPt_0_new[t]=P0/W0;
    DPt_1_new[t]=P1/W1;
    DLt_0_new[t]=L0/W0;
    DLt_1_new[t]=L1/W1;

    summ1=MLmax/(NO+t);
    MDnew=MDnew+summ1;
    MDt_new[t]=summ1;

    for(int i=0;i<NS;i++)PSnew[t][i]=Py[i];

  }


  //////////

  E=-lambda_m*MDnew-lambda_p*DPnew+lambda_c*DCnew;

  if(E < E_opt){

    E_opt=E;
    TR=TRnew;
    TW=TWnew;

    DP=DPnew;
    DC=DCnew;
    MD=MDnew;

    for(int t=ti;t<T+1;t++){
      i0=seq[t];
      tim[i0]=NS;
    }

    for(int t=ti;t<T+1;t++){
      i0=seq_new[t];
      seq[t]=i0;
      tim[i0]=t;
      MDt[t]=MDt_new[t];
      DPt[t]=DPt_new[t];
      DCt[t]=DCt_new[t];
      DLt[t]=DLt_new[t];
      DPt_0[t]=DPt_0_new[t];
      DPt_1[t]=DPt_1_new[t];
      DLt_0[t]=DLt_0_new[t];
      DLt_1[t]=DLt_1_new[t];
```

```
    for(int a=0;a<ND;a++)PD[t][a]=PDnew[t][a];
    for(int i=0;i<NS;i++)PS[t][i]=PSnew[t][i];
  }
  report();

}else{

  for(int i=0;i<NS;i++)So[i]=Sold[i];

}



}
/////////////////// update_seq




////////////////////////////////////////////////////////////////
MC ////////////////////////////////////////////////////////////////
//////////




////////////////////// initial_D

void initial_D()
{


for(int a=0;a<ND;a++){
  D[a]=0;
  if(rang(seed)<float(M)/ND)D[a]=1;
}

///

ML=compute_ML();

MLmax=ML;
for(int a=0;a<ND;a++)Dmax[a]=D[a];


}
////////////////////// end initial_D




////////////////////// compute_ML

double compute_ML()
```

```
{

int a,b,c;
int la,lc;
double summ,f;



f=0;
for(int a=0;a<ND;a++)f=f+Ka[a]*D[a];

///

for(int i=0;i<NS;i++){
       if(So[i]!=0){
         summ=Ki[i];
         for(int li=0;li<kia[i];li++){
           a=via[i][li];
           la=vial[i][li];
           summ += Kai[a][la]*D[a];
         }
         for(int li=0;li<kic[i];li++){
           c=vic[i][li];
           lc=vicl[i][li];
           a=vca[c][0];
           b=vca[c][1];
           summ += Kci[c][lc]*D[a]*D[b];
         }
         f=f+summ*So[i]-log(2*cosh(summ));
      }
}

///

return f;

}
////////////////////// end compute_ML




////////////////////// compute_hi

double compute_hi(int i)
{

int a,b,c;
int la,lc;
double hi;


       hi=Ki[i];
       for(int li=0;li<kia[i];li++){
```

```
      a=via[i][li];
      la=vial[i][li];
      hi += Kai[a][la]*D[a];
    }
    for(int li=0;li<kic[i];li++){
      c=vic[i][li];
      lc=vicl[i][li];
      a=vca[c][0];
      b=vca[c][1];
      hi += Kci[c][lc]*D[a]*D[b];
    }


return hi;

}
////////////////////// end compute_hi




////////////////////// compute_dL

double compute_dL(int ao)
{

int a,b,c;
int la,lc;
double dL;
double summ,dsumm;


    dL=Ka[ao]*(1-2*D[ao]);
    for(int i=0;i<NS;i++){
       if(So[i]!=0){
       summ=Ki[i];
       dsumm=0;
       for(int li=0;li<kia[i];li++){
         a=via[i][li];
         la=vial[i][li];
         summ += Kai[a][la]*D[a] ;
         if(a==ao)dsumm += Kai[a][la]*(1-2*D[a]);
       }
       for(int li=0;li<kic[i];li++){
         c=vic[i][li];
         lc=vicl[i][li];
         a=vca[c][0];
         b=vca[c][1];
         summ += Kci[c][lc]*D[a]*D[b];
         if(a==ao)dsumm += Kci[c][lc]*(1-2*D[a])*D[b];
         if(b==ao)dsumm += Kci[c][lc]*(1-2*D[b])*D[a];
       }
       dL=dL+dsumm*So[i]+log(2*cosh(summ))-log(2*cosh(summ+dsumm));
     }
```

```
    }

    return dL;

}
////////////////////// end compute_dL




////////////////////// compute_PDS

void compute_PDS()
{


int ao,to;
int Nav;
int dt=20;
double dL;
double hi;


for(int a=0;a<ND;a++)Px[a]=0;

for(int i=0;i<NS;i++){
  Py[i]=0.0;
  if(So[i]!=0)Py[i]=(1.0+So[i])/2.0;
}

////////////


Nav=0;
to=0;
for(int t=0;t<tmax_MC;t++){
  to=to+1;

  make_O(ND);

///

  for(int l=0;l<ND;l++){
    ao=O[l];

    dL=compute_dL(ao);

     if(rang(seed)<exp(dL)){
         ML=ML+dL;
         D[ao]=1-D[ao];
         if(ML>MLmax){
             MLmax=ML;
             for(int a=0;a<ND;a++)Dmax[a]=D[a];
         }
```

```
     }

  }

///

  if((t>tmax_EQ)&&(to>dt)){
      to=0;
      Nav=Nav+1;
      for(int a=0;a<ND;a++)Px[a]=Px[a]+D[a];
      for(int i=0;i<NS;i++){
          if(So[i]==0){
            hi=compute_hi(i);
            Py[i]=Py[i]+(1.0+tanh(hi))/2.0;
          }
      }
  }

///
}


////////////

for(int a=0;a<ND;a++){
Px[a]=Px[a]/Nav;
}

for(int i=0;i<NS;i++){
if(So[i]==0)Py[i]=Py[i]/Nav;
}


}
////////////////////// end compute_PDS






////////////////////////////////////////////////////////////////////
Dmax ///////////////////////////////////////////////////////////////
////////////




////////////////////// compute_Dmax

void compute_Dmax()
{

int ao;
```

```
double dL;
double beta,dbeta;


beta=1.0;
dbeta=9.0/tmax_ML;
for(int t=0;t<tmax_ML;t++){

  make_O(ND);

  for(int l=0;l<ND;l++){
     ao=O[l];

     dL=compute_dL(ao);

     if(rang(seed)<exp(beta*dL)){
         ML=ML+dL;
         D[ao]=1-D[ao];
         if(ML>MLmax){
             MLmax=ML;
             for(int a=0;a<ND;a++)Dmax[a]=D[a];
         }
     }

  }


beta=beta+dbeta;
}


}
////////////////////// end compute_Dmax





////////////////////// compute_E

void compute_E()
{

int to;
int imax;
int cr,cw;
double summ1,summ2;
double W0,W1,P0,P1,L0,L1;


cr=0;
cw=0;
TR=T+1;
TW=T+1;
```

```
for(int i=0;i<NS;i++){
  if(tim[i]>0)So[i]=0;
}

///////

initial_D();
compute_PDS();
compute_Dmax();


for(int a=0;a<ND;a++)PD[0][a]=Px[a];
for(int i=0;i<NS;i++)PS[0][i]=Py[i];

P0=0;
P1=0;
L0=0;
L1=0;
W0=0;
W1=0;
summ1=0;
summ2=0;
for(int a=0;a<ND;a++){
    summ1=summ1+weight[a]*fabs(Px[a]-0.5);
    summ2=summ2+weight[a]*(Px[a]-0.5)*(2*xd[a]-1);
    if(xd[a]==0){
        W0=W0+weight[a];
        P0=P0+weight[a]*fabs(Px[a]-0.5);
        L0=L0+weight[a]*(Px[a]-0.5)*(2*xd[a]-1);
    }else{
        W1=W1+weight[a];
        P1=P1+weight[a]*fabs(Px[a]-0.5);
        L1=L1+weight[a]*(Px[a]-0.5)*(2*xd[a]-1);
    }
    if((xd[a]==1)&&(Px[a]>Pth)&&(cr==0)){
        cr=1;
        TR=0;
    }
    if((xd[a]==0)&&(Px[a]>Pth)&&(cw==0)){
        cw=1;
        TW=0;
    }
}
DPt[0]=summ1/W;
DPt_0[0]=P0/W0;
DPt_1[0]=P1/W1;
DLt[0]=summ2/W;
DLt_0[0]=L0/W0;
DLt_1[0]=L1/W1;
MDt[0]=MLmax/NO;
DCt[0]=0;
seq[0]=-1;
```

```
///////


DP=0;
DC=0;
MD=0;
to=1;
while(to<=T){

  imax=seq[to];

  So[imax]=-1;
  if(PS[to-1][imax]>0.5)So[imax]=+1;
  tim[imax]=to;
  DCt[to]=cost[imax];
  seq[to]=imax;

  ///

  initial_D();
  compute_PDS();
  compute_Dmax();

  for(int a=0;a<ND;a++)PD[to][a]=Px[a];
  for(int i=0;i<NS;i++)PS[to][i]=Py[i];

  P0=0;
  P1=0;
  L0=0;
  L1=0;
  W0=0;
  W1=0;
  summ1=0;
  summ2=0;
  for(int a=0;a<ND;a++){
    summ1=summ1+weight[a]*fabs(Px[a]-0.5);
    summ2=summ2+weight[a]*(Px[a]-0.5)*(2*xd[a]-1);
    if(xd[a]==0){
        W0=W0+weight[a];
        P0=P0+weight[a]*fabs(Px[a]-0.5);
        L0=L0+weight[a]*(Px[a]-0.5)*(2*xd[a]-1);
    }else{
        W1=W1+weight[a];
        P1=P1+weight[a]*fabs(Px[a]-0.5);
        L1=L1+weight[a]*(Px[a]-0.5)*(2*xd[a]-1);
    }
    if((xd[a]==1)&&(Px[a]>Pth)&&(cr==0)){
        cr=1;
        TR=to;
    }
    if((xd[a]==0)&&(Px[a]>Pth)&&(cw==0)){
        cw=1;
```

```
        TW=to;
      }
    }
    DPt[to]=summ1/W;
    DPt_0[to]=P0/W0;
    DPt_1[to]=P1/W1;
    DLt[to]=summ2/W;
    DLt_0[to]=L0/W0;
    DLt_1[to]=L1/W1;
    MDt[to]=MLmax/(NO+to);

    DP=DP+DPt[to];
    DC=DC+DCt[to];
    MD=MD+MDt[to];


    ///

    to=to+1;

  }


  }
  ////////////////////// end compute_E





  ////////////////////////////////////////////////////////////////////
  ////////////////////////////////////////////////////////////////////
  ///////////





  /////////////////// report
  void report()
  {


  ofstream output("RW.dat");
  output<<"#t"<<" "<<"observation sequence"<<" "<<"log-likelihood
  ML(t)"<<" "<<"polarization DP(t)"<<" "<<"sign cost SC(t)"<<" "<<"
  overlap with true disease pattern DL(t)"<<" "<<"first right
  diagnosis time T_R"<<" "<<"first wrong diagnosis time T_W"<<endl;


  for(int t=0;t<T+1;t++){
  output<<t<<" "<<seq[t]<<" "<<MDt[t]<<" "<<DPt[t]<<" "<<DCt[t]<<"
  "<<DLt[t]<<" "<<TR<<" "<<TW<<endl;
  }
```

```
}
/////////////////// report




////////////////////// rang

double rang(int& seed)
{

      int a, m, q, r, l;
      double conv, rand;


      a = 16807;
      m = 2147483647;
      q = 127773;
      r = 2836;

      conv = 1.0 / (m - 1);

      l = seed / q;
      seed = a * (seed - q * l) - r * l;
      if (seed < 0) {
              seed += m;
      }
      rand = conv * (seed - 1);



      return rand;
}
////////////////////// end rang
```
